# Diterpenoids with Schistosomula-Killing and Anti-Fibrosis Activities In Vitro from the Leaves of *Croton tiglium*

**DOI:** 10.3390/molecules29020401

**Published:** 2024-01-13

**Authors:** Li Li, Biqing Zhao, Xiaoxiao Zheng, Zhaohui Liu, Huan Zou, Li Qin, Xiaojiang Zhou

**Affiliations:** 1 College of Pharmacy, Hunan University of Chinese Medicine, Changsha 410208, China; lili19950711@126.com (L.L.); qingerhn@126.com (B.Z.); xiao1320329479@163.com (X.Z.); 2 Hengxiu Tang Pharmaceutical Co., Ltd., Changsha 410219, China; liuzhaohui@yfpharmacy.com (Z.L.); zouhuan@yfpharmacy.com (H.Z.)

**Keywords:** diterpenoids, the leaves of *Croton tiglium*, schistosomula killing, anti-fibrosis, TGF-β/Smad pathway

## Abstract

The leaves of *C. tiglium* have been comprehensively researched for their structurally novel bioactive natural compounds, especially those with anti-schistosomiasis liver fibrosis activity, because ethyl acetate extract, which can be extracted from the leaves of *C. tiglium*, has good anti-schistosomiasis liver fibrosis effects. One new tigliane-type diterpene, 20-acetyl-13-*O*-(2-metyl)butyryl-phorbol (**1**), and nine known (**2**–**10**) analogues were isolated from the leaves of *C. tiglium*. Their structures were elucidated on the basis of spectroscopic analysis and ECD analysis. All diterpenoids had a stronger insecticidal effect on schistosomula, and compounds **2**, **4**, and **10** had good anti-liver-fibrosis effects. Furthermore, compared with the model group, compound **2** significantly downregulated the protein and mRNA expression of COL-I, COL-III, α-SMA, and TGF-β1 on TGF-β1-induced liver fibrosis in LX-2 cells. Meanwhile, compound **2** also regulated the expression of TGF-β/Smad-pathway-related proteins. The results suggest that diterpenoids from *C. tiglium* may serve as potential schistosomula-killing and anti-liver-fibrosis agents in the future.

## 1. Introduction

Schistosomiasis is a zoonosis caused by Schistosoma parasites that is widely prevalent in tropical and subtropical areas and has drawn limited research attention [1,2]. There are multiple types of schistosomes, mainly including *Schistosoma aegypti*, *S. mansoni*, and *S. japonicum* [3]. Schistosomiasis affects more than 70 countries or regions worldwide. According to statistics from the World Health Organization (WHO), at least 240 million people worldwide are infected with schistosomiasis, and over 800 million people live in high-risk areas [1,4]. Schistosomiasis has a long history of prevalence in China. Typical eggs of *S. japonicum* were found in the Western Han female corpses in Mawangdui, Changsha, Hunan, and in the Western Han male corpses in Jiangling, Hubei. This confirms that as early as 2100 years ago, there was an epidemic of *S. japonicum* in China [5]. As of the end of 2020, there were a total of 450 schistosomiasis-endemic counties (cities, districts) in China, 3352 schistosomiasis-endemic townships (towns), 28,376 endemic villages, and a total population of 71.3704 million in endemic villages [6].

Schistosomiasis is an immune disease that can cause damage to the human body during its infection process, including cercariae, schistosomula, adults, and eggs. The main reason for the damage is that antigens released at different stages of the worm’s life can induce a series of immune pathological changes and some complications [7]. The biggest damage is secondary fibrosis caused by Schistosoma cercariae eggs deposited in the liver [8,9]. TGF-β_1_ is currently recognized as a pro-fibrotic factor, mainly acting through the phosphorylation of smad2/3 in the smad protein family. It has the effects of activating hepatic stellate cells, promoting collagen synthesis, and ultimately leading to liver fibrosis [10]. There have been multiple reports on the relationship between liver fibrosis in schistosomiasis and TGF in hepatic stellate cells-β_1_/Smads signal transduction [11,12]. 

At present, the preferred drug for treating schistosomiasis is praziquantel, which has good therapeutic effects. In addition to directly acting on schistosomiasis, praziquantel also has a certain degree of immune dependence and immune synergy, which can reduce the degree of liver fibrosis but still cannot change the fibrosis process or reverse liver fibrosis [13,14,15]. In addition, praziquantel has some mild side effects such as headache, nausea, anorexia, and reports of drug resistance [16,17]. The research on schistosome vaccines has been going on for over half a century, but vaccines obtained through traditional methods have unsatisfactory or unstable immune protection against human induction. Recombinant vaccines using genetic engineering technology are either in the laboratory research stage or in the clinical trial stage, and there is still a certain distance from practical application [18]. Chinese herbs, which are essential components of global traditional medicine, have been widely used for the treatment of schistosomiasis, such as artemisinin and its derivatives, which have become drugs for the prevention and early treatment of schistosomiasis. More importantly, Chinese herbs have unique advantages in treating liver fibrosis caused by schistosomiasis [19,20]. Therefore, the search for new anti-schistosomiasis liver fibrosis drugs in Chinese herbs is a research hotspot.

The seeds and leaves of *Croton tiglium*, which belongs to the Euphorbiaceae family, are known as traditional medicinal plants in China [21,22]. In the early stage, we found a series of active diterpenoids against tumor from *C. tiglium* and that the ethyl acetate extract of the leaves of *C. tiglium* has good anti-schistosomiasis liver fibrosis effects [23,24,25,26]. As part of our ongoing efforts to discover structurally novel bioactive natural compounds, especially those with anti-schistosomiasis liver fibrosis activity, in *C. tiglium*, we re-examined the chemical constituents of the leaves of *C. tiglium*. One new tigliane-type diterpene (**1**), together with nine known analogues were isolated from the ethyl acetate extract of *C. tiglium*. Herein, we report the in vitro isolation, structural elucidation, and schistosomula-killing and anti-fibrosis activities of these compounds.

## 2. Results and Discussion

### 2.1. Structural Elucidation of Compound ***1***

**Figure 1:**
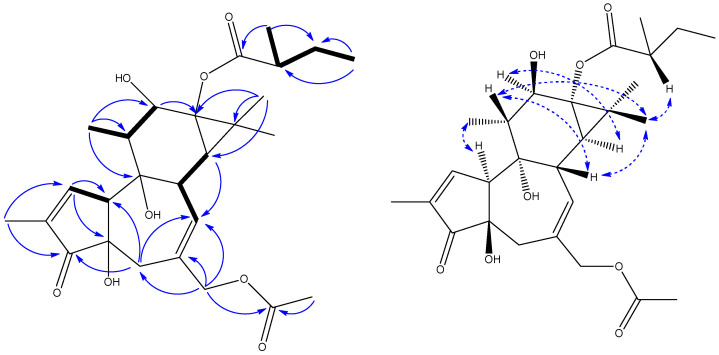
The ^1^H-^1^H COSY (bold), HMBC (arrows), and key ROESY (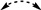) correlations of **1**.

**Figure 2:**
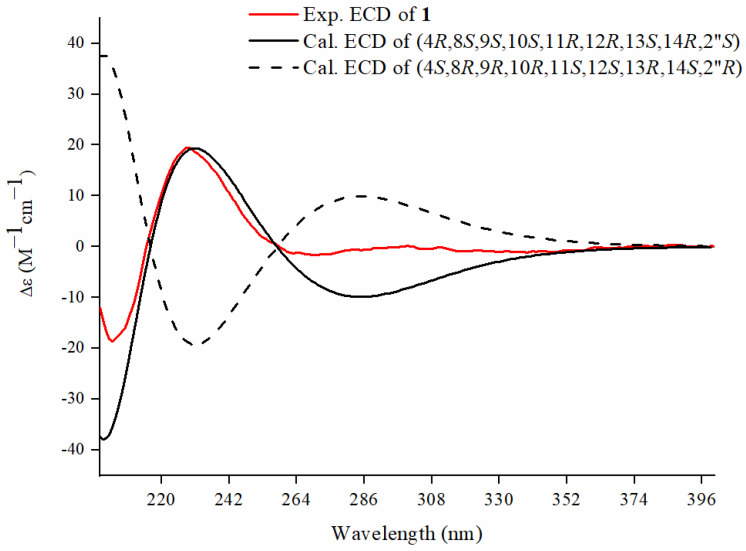
Experimental and calculated ECD spectra of **1**.

**Figure 3:**
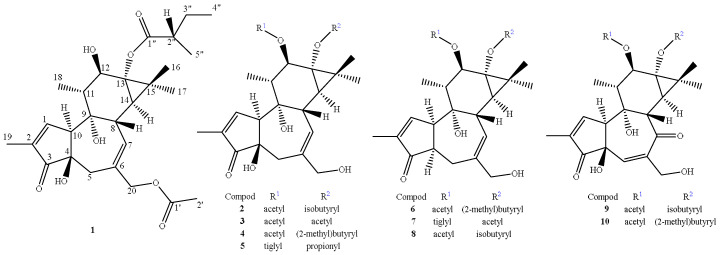
Structures of compounds **1**–**10**.

Compound **1** was obtained as a colorless oil. Its molecular formula, C_27_H_38_O_8_, was deduced from the [M + H]^+^ ion at *m*/*z* 491.2639 (calcd 491.2630) in the high-resolution electrospray ionization mass spectrometry (HRESIMS) spectrum, which was in accordance with the ^1^H and ^13^C nuclear magnetic resonance (NMR) spectroscopic data (Table 1), corresponding to nine degrees of unsaturation. The infrared (IR) spectrum showed the existence of a hydroxyl group (3334 cm^−1^) and double-bond group (1653 cm^−1^). In the ^1^H NMR spectrum, the most salient signals were for two olefinic protons at *δ*_H_ 7.60 (s) and 5.72 (1H, dd, *J* = 5.5, 1.7 Hz), one oxygen-bearing methylene proton at *δ*_H_ 4.50 (s), one oxygen-bearing methine proton at *δ*_H_ 3.89 (1H, d, *J* = 9.8 Hz), and seven methyl resonances at *δ*_H_ 2.04 (3H, s), 1.77 (3H, dd, *J* = 2.7, 1.2 Hz), 1.27 (3H, s), 1.26 (3H, s), 1.20 (3H, d, *J* = 7.0 Hz), 1.09 (3H, d, *J* = 6.5 Hz), and 0.98 (3H, t, *J* = 7.4 Hz). The ^13^C NMR (Table 1), distortionless enhancement by polarization transfer (DEPT), and heteronuclear single quantum coherence (HSQC) spectra revealed the presence of 27 carbons including three carbonyl carbons at *δ*_C_ 208.8, 179.8, and 171.2; two olefinic quaternary carbons at *δ*_C_ 136.2 and 133.1; three oxygen-bearing quaternary carbons at *δ*_C_ 78.2, 73.1, and 67.5; one quaternary carbon at *δ*_C_ 25.8; two aromatic unsaturated methine carbons at *δ*_C_ 159.4 and 132.5; one oxygen-bearing methine carbon at *δ*_C_ 76.2; five methine carbons at *δ*_C_ 56.2, 44.9, 41.1, 39.0, and 35.2; one oxygen-bearing methylene carbon at *δ*_C_ 69.2; two methylene carbons at *δ*_C_ 37.6 and 26.2; and a set of signals attributable to seven methyl groups at *δ*_C_ 22.9, 19.4, 16.0, 15.5, 14.1, 10.6, and 8.8. The ^1^H–^1^H correlated spectroscopy (COSY) correlations (Figure 1) indicated the presence of H-1/H-10, H-7/H-8/H-14, H_3_-18/H-11/H-12, and H_3_-5″/H-2″/H_2_-3″/H_3_-4″ moieties. Furthermore, a set of key correlations from H_3_-19 to C-1 (*δ*_C_ 159.4), C-3 (*δ*_C_ 208.8); H_2_-5 to C-3 (*δ*_C_ 208.8), C-7 (*δ*_C_ 132.5), C-10 (*δ*_C_ 56.2); H_2_-20 to C-5 (*δ*_C_ 37.6), C-6 (*δ*_C_ 136.2), C-7 (*δ*_C_ 132.5); H_3_-18 to C-9 (*δ*_C_ 78.2), C-11 (*δ*_C_ 44.9), C-12 (*δ*_C_ 76.2); H_3_-16 to C-13 (*δ*_C_ 67.5), C-14 (*δ*_C_ 35.2), C-15 (*δ*_C_ 25.8); H_3_-2′ to C-1′ (*δ*_C_ 171.2); H_3_-5″ to C-1″ (*δ*_C_ 179.8), C-3″ (*δ*_C_ 26.2); and H_3_-4″ to C-2″ (*δ*_C_ 41.1), C-3″ (*δ*_C_ 26.2) are presented in heteronuclear multiple-bond correlations (HMBCs) (Figure 1). These features characteristically revealed the structure of **1** as possessing a tigliane (phorbol) backbone, consistent with the known compounds **2**–**5** and 13-*O*-(2-metyl)butyryl-phorbol [23,24,27,28,29]. The ^1^H and ^13^C NMR spectra of **1** were closely related to those of 13-*O*-(2-metyl)butyryl-phorbol [23]. Comparison of their NMR data revealed that one acetyl group signal (171.2, 2.04/19.4) was observed in **1**. The key HMBC correlation between H_2_-20 and C-1′ (*δ*_C_ 171.2) indicated that this acetyl group was connected to C-20 via an ester bond (Figure 1). The relative configuration of compound **1** was deduced from ROESY correlations and comparison with data reported in the literature. The ROESY correlations of H-8/H-11, H-11/H-17, H-17/H-8, and H-17/H_3_-5″ indicated that they are all cofacial, arbitrarily assigned as β-oriented. Meanwhile, the correlation between H-12 and H-14 appeared to be α-oriented (Figure 1). Although no other ROESY correlation could support the trans A/B ring junction with H-10α and OH-4β as well as the 13-(2-methyl)butyryl group in an *α*-position, their orientations were based on biogenetic considerations from all phorbol-type diterpenes characterized so far because the carbon chemical shift values of **1** were in good agreement with those of structurally related compounds [23,24,27,28,29]. To determine its absolute configuration, conformational analyses were carried out via random searching in Sybyl-X 2.0 using the MMFF94S force field with an energy cutoff of 5 kcal/mol. The conformers of **1** were used as the input for the structural optimization by the time-dependent density functional theory (TD-DET) method. The calculated ECD spectrum of (4*R*, 8*S*, 9*S*, 10*S*, 11*R*, 12*R*, 13*S*, 14*R*, 2″*S*)-**1** was consistent with the experimental one (Figure 2), confirming the absolute configuration of **1** to be 4*R*, 8*S*, 9*S*, 10*S*, 11*R*, 12*R*, 13*S*, 14*R,* and 2″*S*, respectively. Therefore, compound **1** was assigned as 20-acetyl-13-*O*-(2-metyl)butyryl-phorbol. The known compounds were identified as 12-*O*-acetylphorbol-13-isobutyrate (**2**) [24], phorbol 12,13-diacetate (**3**) [27], 12-*O*-acetylphorbol-13-(2″-methyl)butyrate (**4**) [28], 12-*O*-tiglylphorbol-13-propionate (**5**) [29], 12-*O*-acetyl-4α-deoxyphorbol-13-(2″-methyl)butyrate (**6**) [28], 12-*O*-tiglyl-4-deoxy-4α-phorbol-13-acetate (**7**) [22], 4α-deoxyphorbol 12-acetate-13-isobutyrate (**8**) [30], 12-*O*-acetyl-5,6-didehydro-7-oxophorbol-13-yl-2-methylpropanoate (**9**) [31], and 12-*O*-acetyl-5,6-didehydro-7-oxophorbol-13-yl-2-methylbutanoate (**10**) [31], by comparison of our spectroscopic data with those in the literature (Figure 3).

### 2.2. Effect of Schistosomula Killing

Compounds have strong killing effects on schistosomula. Compared with the blank control group, the survival rate of schistosomula in different concentration groups at 24, 48, and 72 h decreased (*p* < 0.05), with the 34.00 μg/mL group having the best killing effect, and after 72 h, the mortality rate of schistosomula in different concentrations of each compound group reached 100%. The results also showed that the survival rate of the Schistosoma cercariae body in 34.00 μg/mL of all compounds was lower than that in the praziquantel group (*p* < 0.05, Table 2), indicating that the diterpenoid components in the leaves of *C. tiglium* had a stronger insecticidal effect on schistosomula compared to the praziquantel group. 

### 2.3. Anti-Fibrosis Activities of Compounds ***2***, ***4***, and ***10*** In Vitro

#### 2.3.1. Cell Cytotoxicity of Compounds **2**, **4**, and **10** on LX-2 Cells

**Figure 4:**
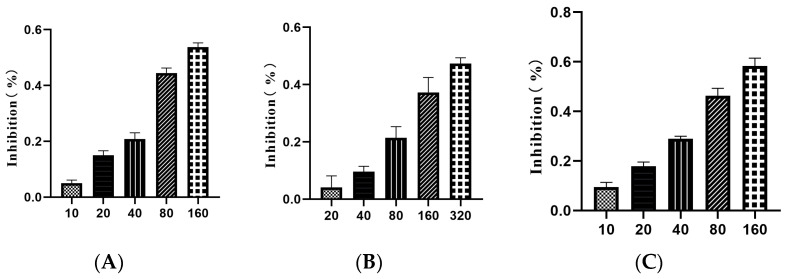
The inhibitory effects of different compounds (**A**) (compound **2**), (**B**) (compound **4**), and (**C**) (compound **10**) on the viability of LX-2 cells.

Compounds **2**, **4**, and **10** showed a certain inhibition effect on the proliferation of LX-2 cells with different concentrations. As the concentration increased, the inhibitory effect of the compounds on the viability of LX-2 cells was enhanced (Figure 4).

According to the calculation results of SPSS (version 26) statistical software, the IC_50_ values of compounds **2**, **4**, and **10** are 103.89, 123.29, and 315.01 µM, respectively, while their TC_0_ values are 2.14, 5.17, and 11.80 µM, respectively (Table 3). Thus, for the low administration group, the concentrations of compounds **2**, **4**, and **10** with an anti-fibrosis effect are set as 0.50, 1.25, and 3.00 µM, respectively; the medium concentrations of compounds **2**, **4**, and **10** are set as 1.00, 2.50, and 6.00 µM; and the high-dose group of compounds **2**, **4**, and **10** are set as 2.00, 5.00, and 12.00 µM, respectively. 

#### 2.3.2. Effect of Compounds **2**, **4**, and **10** on Content of COL-I, COL-III, α-SMA, and TGF-β1 in LX-2 Cells

**Figure 5:**
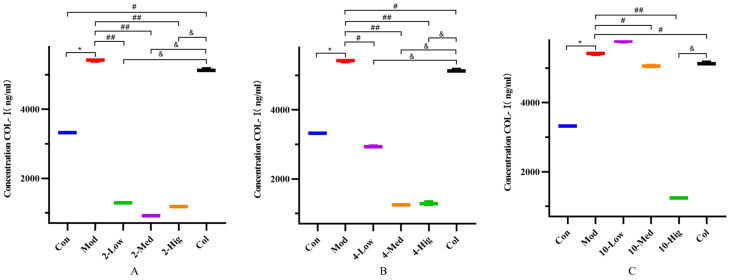
The detection results of COL-I cytokine in supernatant of each group (n = 6). Effect of compounds **2** (**A**), **4** (**B**), and **10** (**C**) on content of COL-I in LX-2 Cells. (Con: blank control group; Mod: model group; Low: low-dose group; Med: middle-dose group; Hig: high-dose group; Col: colchicine group). Compared with the blank control group, * *p* < 0.05; compared with model group, # *p* < 0.05, ## *p* < 0.01; compared with colchicine group, & *p* < 0.05.

**Figure 6:**
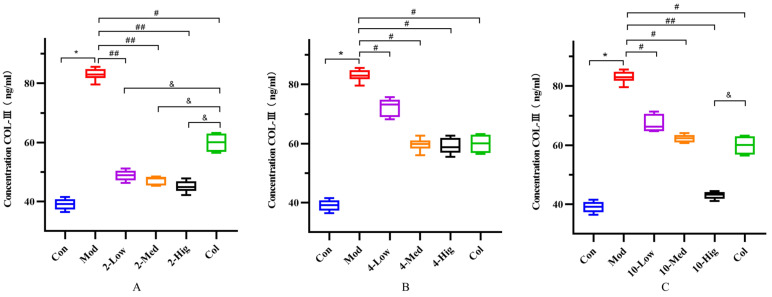
The detection results of COL-III cytokine in supernatant of each group (n = 6). Effect of compounds **2** (**A**), **4** (**B**), and **10** (**C**) on content of COL-III in LX-2 Cells. (Con: blank control group; Mod: model group; Low: low-dose group; Med: middle-dose group; Hig: high-dose group; Col: colchicine group). Compared with the blank control group, * *p* < 0.05; compared with model group, # *p* < 0.05, ## *p* < 0.01; compared with colchicine group, & *p* < 0.05.

**Figure 7:**
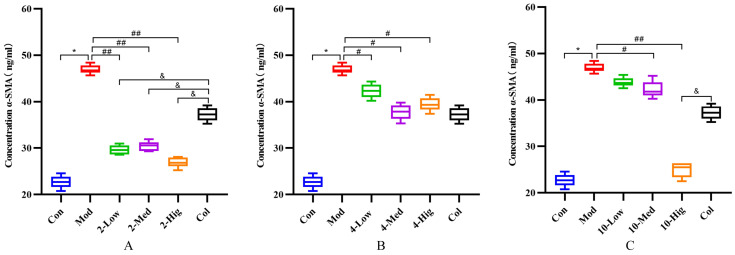
The detection results of α-SMA cytokine in supernatant of each group (n = 6). Effect of compounds **2** (**A**), **4** (**B**), and **10** (**C**) on content of α-SMA in LX-2 Cells. (Con: blank control group; Mod: model group; Low: low-dose group; Med: middle-dose group; Hig: high-dose group; Col: colchicine group). Compared with the blank control group, * *p* < 0.05; compared with model group, # *p* < 0.05, ## *p* < 0.01; compared with colchicine group, & *p* < 0.05.

**Figure 8:**
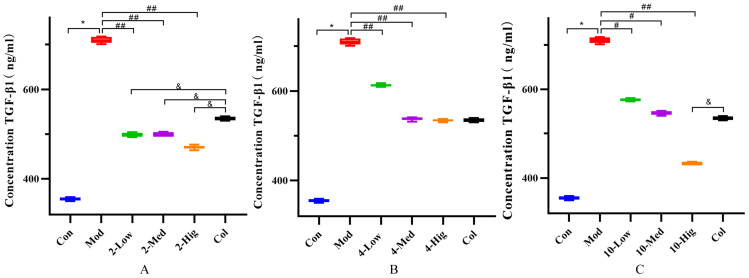
The detection results of TGF-β1 cytokine in supernatant of each group (n = 6). Effect of compounds **2** (**A**), **4** (**B**), and **10** (**C**) on content of TGF-β1 in LX-2 Cells. (Con: blank control group; Mod: model group; Low: low-dose group; Med: middle-dose group; Hig: high-dose group; Col: colchicine group). Compared with the blank control group, * *p* < 0.05; compared with model group, # *p* < 0.05, ## *p* < 0.01; compared with colchicine group, & *p* < 0.05.

Compared with the blank group, the contents of COL-I, COL-III, α-SMA, and TGF-β1 in the TGF-β1-treated model group were significantly increased, which indicated that the TGF-β1 model was successfully established (Figure 5, Figure 6, Figure 7 and Figure 8). Compared with the model group, compound **2** significantly decreased the contents of COL-I, COL-III α-SMA, and TGF-β1 (*p* < 0.01); compound **4** decreased the contents of them (*p* < 0.05); and compound **10** decreased the contents of α-SMA and TGF-β1 in each dose group (*p* < 0.05, Figure 5, Figure 6, Figure 7 and Figure 8). Based on the above results, compound **2** is selected for the next research on inhibiting liver fibrosis.

#### 2.3.3. Inhibitory Effects of Compound **2** on TGF-β1-Induced Liver Fibrosis in LX-2 Cells

##### The mRNA Expression of COL-I, COL-III, α-SMA, TGF-β1 in LX-2 Cells

**Figure 9:**
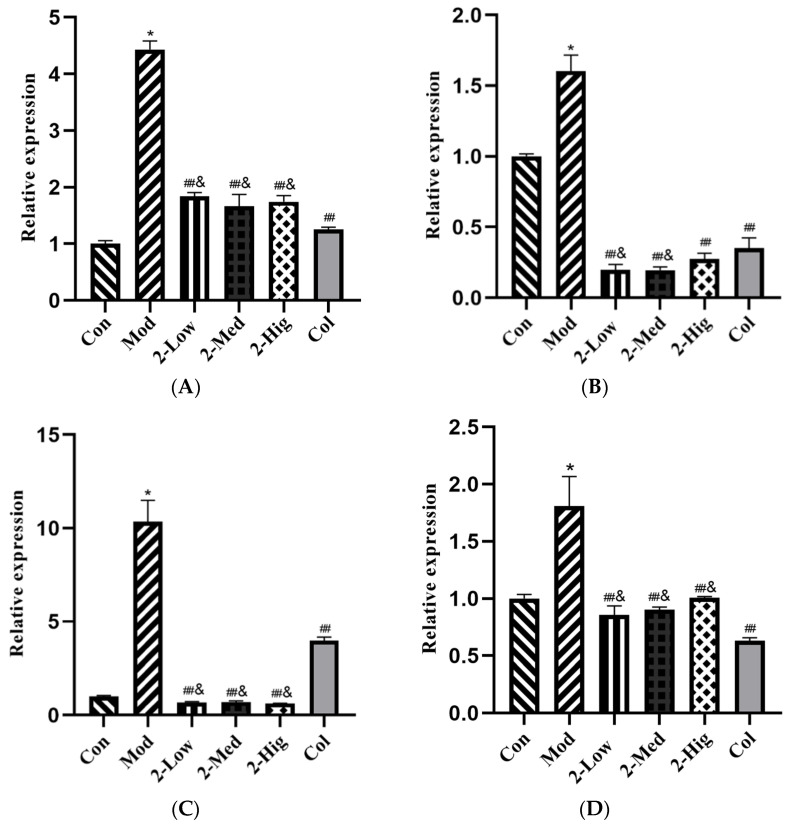
COL-I (**A**), COL-III (**B**), α- SMA (**C**), and TGF-β 1 (**D**) mRNA expression levels (n = 3) (Con: blank control group; Mod: model group; Low: low-dose group; Med: middle-dose group; Hig: high-dose group; Col: colchicine group). Compared with the blank control group, * *p* < 0.05; compared with model group, ## *p* < 0.01; compared with colchicine group, & *p* < 0.05.

The results showed that compared with the blank group, the mRNA expression of COL-I, COL-III, α-SMA, and TGF-β1 was upregulated in the model group (*p* < 0.05). Compared with the model group, the different dosage groups of compound **2** significantly downregulated the mRNA expression levels of COL-I, COL-III, α-SMA, and TGF-β1 (*p* < 0.01). Compared with the colchicine group, the different dosage groups of compound **2** downregulated the mRNA expression levels of COL-III and α-SMA at a lower level (*p* < 0.05) (Figure 9).

##### The Protein Expression of COL-I, COL-III, α-SMA, and TGF-β1 in LX-2 Cells

**Figure 10:**
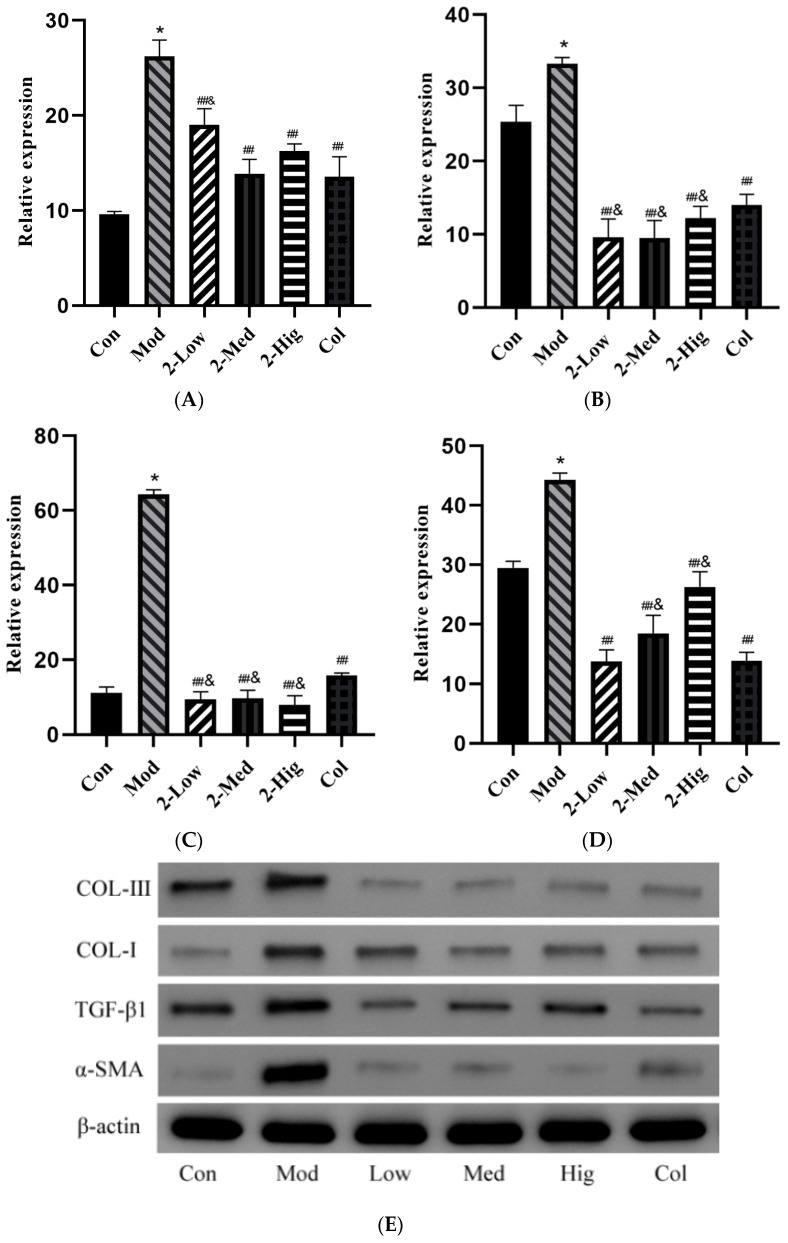
Relative expression of COL-I, COL-III, α-SMA, and TGF-β1 proteins versus β-actin in LX-2 cells (**A**–**D**); detected expression of COL-I, COL-III, α-SMA, TGF-β1, and β-actin proteins in LX-2 cells using WB (**E**) (n = 3) (Con: blank control group; Mod: model group; Low: low-dose group; Med: middle-dose group; Hig: high-dose group; Col: colchicine group). Compared with the blank control group, * *p* < 0.05; compared with model group, ## *p* < 0.01; compared with colchicine group, & *p* < 0.05.

As shown in Figure 10, compared with the blank group, the protein expression levels of COL-I, COL-III, α-SMA, and TGF-β1 were upregulated in the model group (*p* < 0.05). Compared with the model group, the different dosage groups of compound **2** significantly downregulated the protein expression of COL-I, COL-III, α-SMA, and TGF-β1 (*p* < 0.01). Compared with the colchicine group, the different dosage groups of compound **2** downregulated the protein expression levels of COL-III and α-SMA at a lower level (*p* < 0.05).

##### The Expression of COL-I, COL-III, α-SMA, and TGF-β1 in LX-2 Cells Were Evaluated Using Immunohistochemistry Staining 

**Figure 11:**
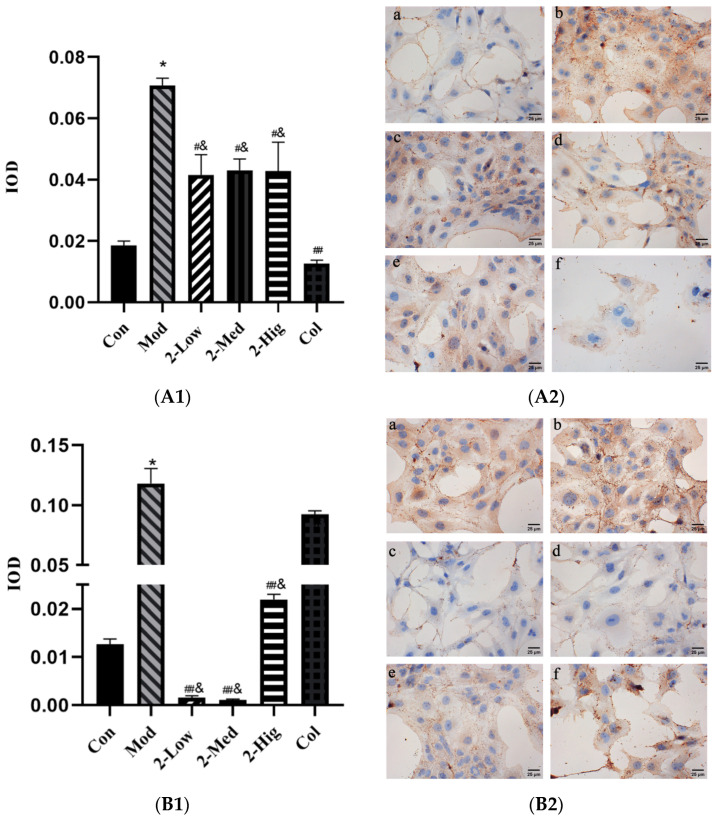

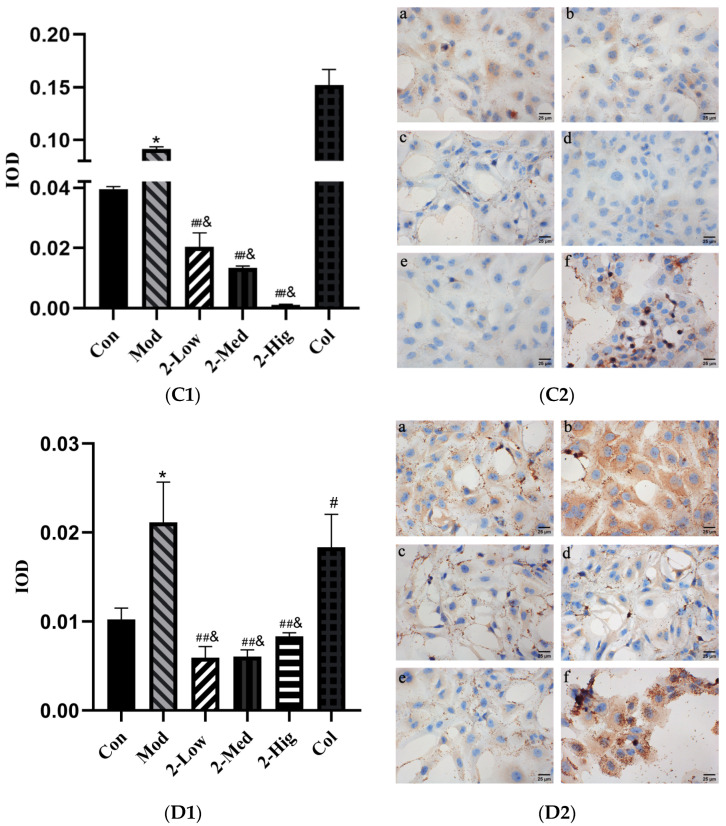
(**A1**–**D2**) The expression of COL-I, COL-III, α-SMA, and TGF-β1 measured using immunohistochemistry staining (n = 3) [Con: blank control group (**a**); Mod: model group (**b**); Low: low-dose group (**c**); Med: middle-dose group (**d**); Hig: high-dose group (**e**); Col: colchicine group (**f**)]. Compared with the blank control group, * *p* < 0.05; compared with model group, # *p* < 0.05, ## *p* < 0.01; compared with colchicine group, & *p* < 0.05.

The immunohistochemical results showed that compared with the blank group, the expression levels of COL-I, COL-III, α-SMA, and TGF-β1 were significantly upregulated in the model group (*p* < 0.05). Compared with the model group, the expression level of COL-I was downregulated (*p* < 0.05), while those of COL-III, α-SMA, and TGF-β1 were significantly downregulated (*p* < 0.01) in different dosage groups of compound **2** (Figure 11).

#### 2.3.4. Inhibitory Effects of Compound **2** on Liver Fibrosis through the Regulation of the Expression of TGF-β/Smad-Pathway-Related Proteins

##### The mRNA Expression of TGF-βRI, TGF-βRII, Smad2, and Smad3

**Figure 12:**
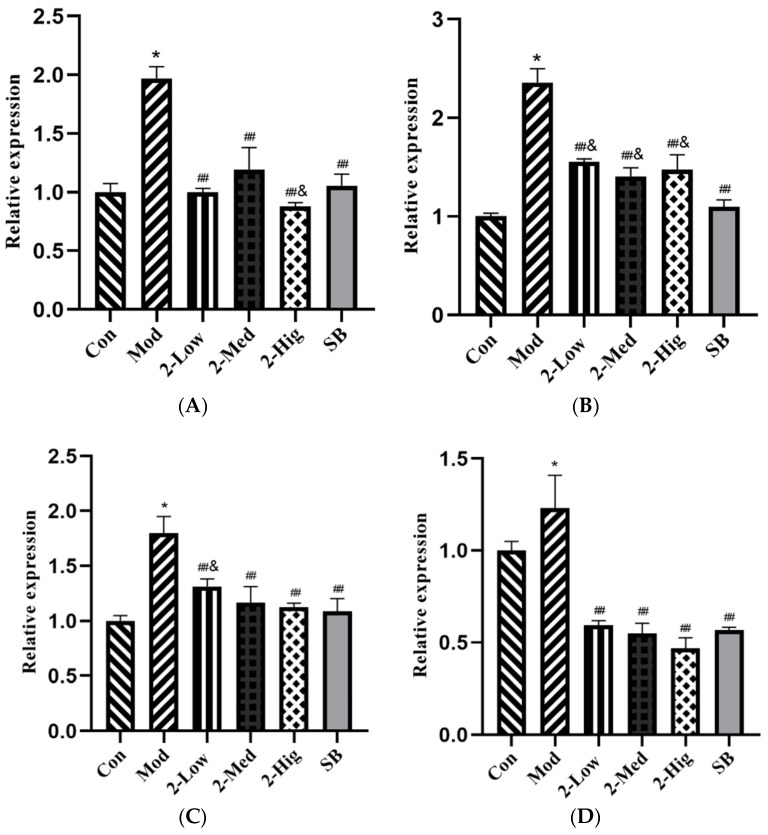
The mRNA expression of TGF-βRI (**A**), TGF-βRII (**B**), Smad2 (**C**), and Smad3 (**D**) was evaluated using RT-PCR (n = 3) (Con: blank control group; Mod: model group; Low: low-dose group; Med: middle-dose group; Hig: high-dose group; SB: SB431542). Compared with the blank control group, * *p* < 0.05; compared with model group, ## *p* < 0.01; compared with colchicine group, & *p* < 0.05.

Compared with the blank group, the mRNA expressions of TGF-βRI, TGF-βRII, Smad2, and Smad3 were increased in the model group that was established by stimulating LX-2 cells with TGF-β1 (*p* < 0.05). Compared with the model group, compound **2** at different doses and SB431542 significantly reduced the mRNA expressions of TGF-βRI, TGF-βRII, Smad2, and Smad3 (*p* < 0.01) (Figure 12).

##### The Protein Expression of TGF-βRI, TGF-βRII, Smad2, and Smad3

**Figure 13:**
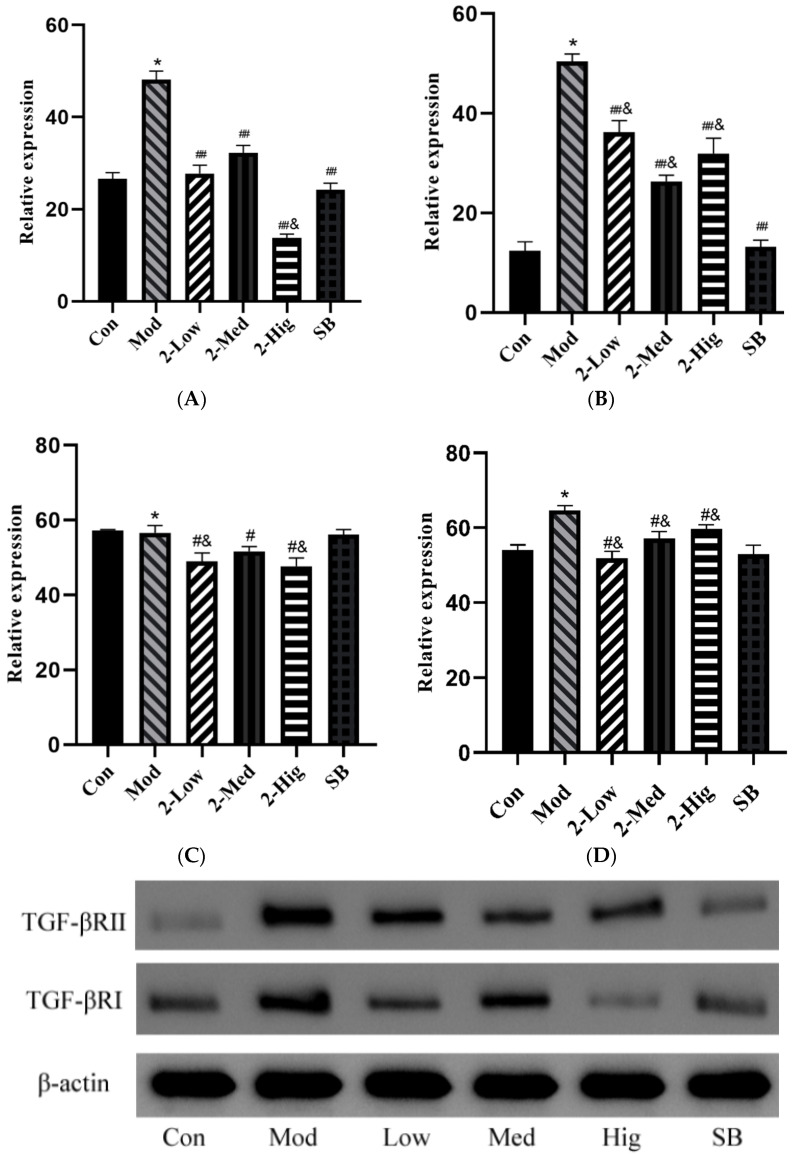

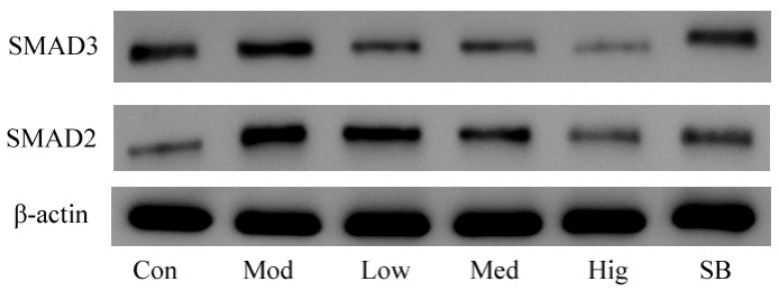
The protein expression of TGF-βRI (**A**), TGF-βRII (**B**), Smad2 (**C**), and Smad3 (**D**) was evaluated by WB (n = 3) (Con: blank control group; Mod: model group; Low: low-dose group; Med: middle-dose group; Hig: high-dose group; SB: SB431542). Compared with the blank control group, * *p* < 0.05; compared with model group, # *p* < 0.05, ## *p* < 0.01; compared with SB431542 group, & *p* < 0.05.

Compared with the blank group, the protein expressions of TGF-βRI, TGF-βRII, Smad2, and Smad3 were upregulated in the model group that was established by stimulating LX-2 cells with TGF-β1 (*p* < 0.05). Compared with the model group, compound **2** at different doses significantly decreased the protein expressions of TGF-βRI and TGF-βRII (*p* < 0.01) and reduced the protein levels of Smad2 and Smad3 (*p* < 0.05) (Figure 13).

##### The Expression of TGF-βRI, TGF-βRII, Smad2, and Smad3 was Evaluated Using Immunohistochemistry Staining 

**Figure 14:**
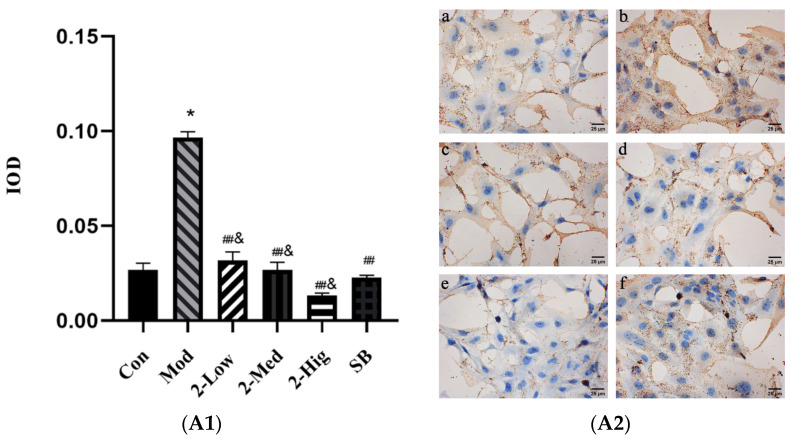

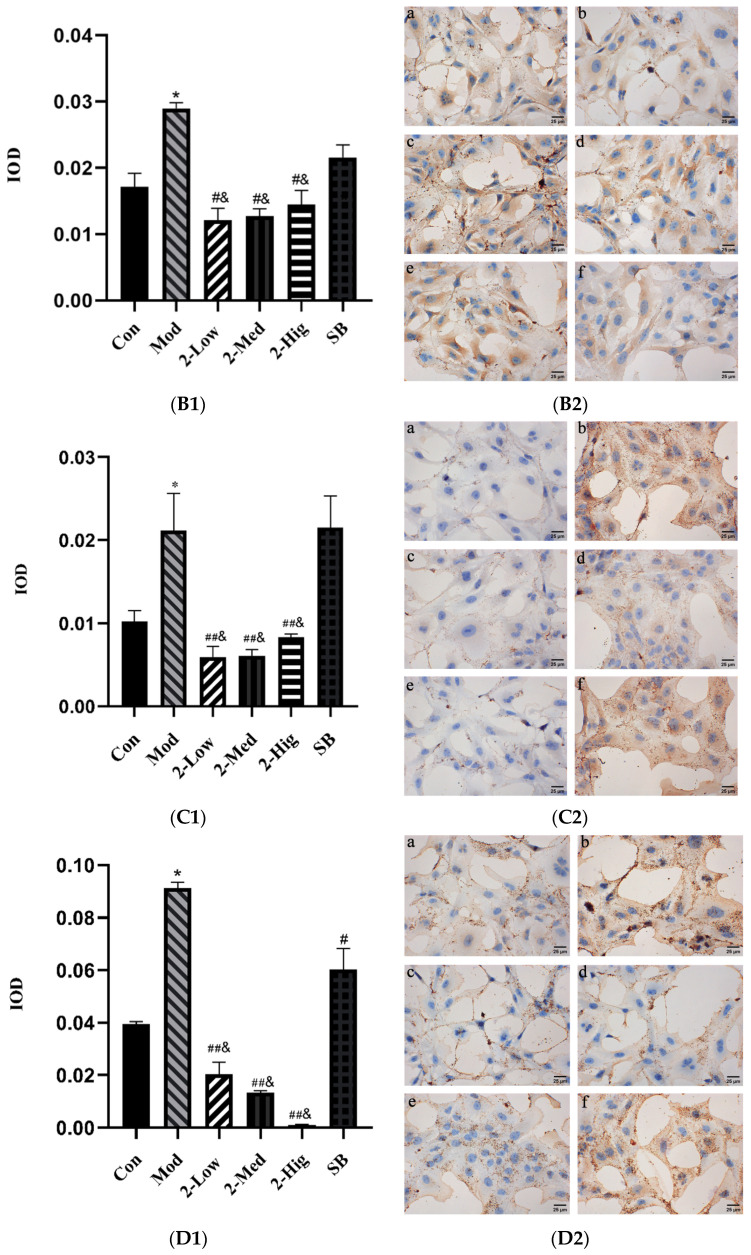
The expression of TGF-βRI (**A1**,**A2**), TGF-βRII (**B1**,**B2**), Smad2 (**C1**,**C2**), and Smad3 (**D1**,**D2**) was evaluated using immunohistochemistry staining (n = 3) [Con: blank control group (**a**); Mod: model group (**b**); Low: low-dose group (**c**); Med: middle-dose group (**d**); Hig: high-dose group (**e**); SB: SB431542 group (**f**)]. Compared with the blank control group, * *p* < 0.05; compared with model group, # *p* < 0.05, ## *p* < 0.01; compared with SB431542 group, & *p* < 0.05.

Compared with the blank group, the expression of TGF-βRI, TGF-βRII, Smad2, and Smad3 was elevated in the model group established by stimulating LX-2 cells with TGF-β1 (*p* < 0.05). Compared with the model group, compound **2** at different doses obviously decreased the expression of TGF-βRI, Smad2, and Smad3 (*p* < 0.01) and reduced the level of TGF-βRII (*p* < 0.05) (Figure 14).

## 3. Materials and Methods

### 3.1. Materials and Reagents 

In August 2020, the leaves of *C. tiglium* were collected from Yibin County in Sichuan Province, China, identified by the corresponding author (X. J. Zhou). A voucher specimen (ZHXJ-0050) was deposited at our laboratory in Hunan University of Chinese Medicine.

NMR spectra were recorded using a Bruker Avance III-600 MHz spectrometer (Bruker BioSpin GmbH, Rheinstetten, Germany). Optical rotations were recorded using a Horiba SEPA-300 polarimeter (Horiba, Tokyo, Japan). IR spectra were obtained using a Tensor 27 (Bruker Optics Gmbh, Ettlingen, Germany) with KBr pellets. HREIMS were determined with an API QSTAR Pulsar 1 spectrometer (MDS Sciex, Concord, ON, Canada). Silica gel (200–300 mesh, Qingdao Marine Chemical Inc., Qingdao, China), RP-18 gel (40–63 µm, Daiso Co., Osaka, Japan), and Sephadex LH-20 (Amersham Biosciences, Uppsala, Sweden) were used for column chromatography. RPMI-1640 culture medium was purchased from Sigma (St. Louis, MO, USA). Pancreatic enzyme digestion solution, PBS, and cell cryopreservation were purchased from abiowell (Changsha, China). Quantitative real-time polymerase chain reaction (QRT-PCR) primers were synthesized by Sangon Biotech Co., Ltd. (Shanghai, China), and the mRNA reverse transcription kit and Ultra-SYBR Mixture were purchased from CoWin Bioscience Co., LTD. (Changsha, China). Semi-preparative HPLC (SHIMADZU LC-10A HPLC system, Reprosil 100 C_18_, 5 µm, 10 × 250 mm) was performed.

### 3.2. Extraction and Isolation

The dried leaves of *C. tiglium* (19 kg) were extracted with 80% MeOH (2 × 90 L) to give a crude extract (1820 g), which was suspended in water and partitioned using petroleum ether and EtOAc (each 6 × 10 L), respectively. The EtOAc extract (470 g) was subjected to column chromatography (CC) over silica gel (200–300 mesh, 7.5 kg) and eluted with a gradient of CHCl_3_/MeOH (100:0, 90:10, 85:15, 80:20, 70:30, 50:50) to give fractions A–C. Fraction A (50.5 g) was divided into six parts (A-1–A-6) using silica gel column eluting with petroleum ether/EtOAc/isopropanol (10:1:0.5). Fraction A-4 (16.4 g) was divided into nine parts (A-4-1–A-4-9) using MCI gel CHP 20P column eluting with gradient aqueous MeOH. Fraction A-4-4 (0.88 g) was submitted by RP-18 gel column (MeOH-H_2_O 2:8-1:0) to obtain compound **10** (15.5 mg). Fraction A-4-7 (2.90 g) was first separated using silica gel column eluting with petroleum ether/EtOAc (1:1) and then divided using semi-preparative HPLC eluting with 56% aqueous acetonitrile to produce compounds **4** (19.7 mg), **7** (4.2 mg), **8** (1.2 mg), and **9** (2.0 mg). Fraction A-4-8 (1.80 g) was first subjected to preparative thin layer chromatography (PTLC) (petroleum ether/EtOAc/isopropanol, 1:1:0.1) and then further purified using Sephadex LH-20 (MeOH) to give compounds **1** (2.5 mg), **2** (9.6 mg), **3** (6.4 mg), **5** (2.2 mg), and **6** (1.2 mg).

### 3.3. Characterization of the Isolates

20-acetyl-13-*O*-(2-metyl)butyryl-phorbol (**1**): colorless oil; [*α*${]}_{D}^{25}$+58.7 (*c* 0.15, MeOH); IR (KBr) *ν*_max_ 3334, 2954, 2833, 1653, 1448, 1018 cm^−1^; ^1^H NMR and ^13^C NMR data, see Table 1; HRESIMS *m*/*z* 491.2639 [M + H]^+^ (calcd for C_27_H_39_O_8_, 491.2630).

The ^1^H NMR, ^13^C NMR, DEPT, HSQC, ^1^H-^1^H COSY, ROESY and HRESIMS spectra of compound **1** are available in the Supplementary Materials.

12-*O*-acetylphorbol-13-isobutyrate (**2**): ^1^H NMR (600 MHz, CD_3_OD): *δ*: 7.55 (1H, br s, H-1), 5.63 (1H, d, *J =* 5.5 Hz, H-7), 5.40 (1H, d, *J =* 10.4 Hz, H-12), 3.97 (1H, d, *J =* 13.0 Hz, H-20α), 3.93 (1H, d, *J =* 13.0 Hz, H-20β), 3.30 (1H, m, H-8), 3.17 (1H, m, H-10), 2.59 (1H, m, H-2″), 2.52 (1H, s, H-5α), 2.50 (1H, s, H-5β), 2.23 (1H, m, H-11), 2.07 (3H, s, H-2′), 1.75 (3H, m, H-19), 1.26 (3H, s, H-16), 1.22 (3H, s, H-17), 1.17 (3H, d, *J =* 4.9 Hz, H-3″), 1.16 (3H, d, *J =* 4.9 Hz, H-4″), 1.10 (1H, d, *J =* 5.3 Hz, H-14), 0.90 (3H, d, *J =* 6.5 Hz, H-18); ^13^C NMR (150 MHz, CD_3_OD): *δ*: 210.3 (C-3), 180.8 (C-1″), 172.8 (C-1′), 160.5 (C-1), 142.8 (C-6), 134.6 (C-2), 129.3 (C-7), 79.7 (C-9), 78.5 (C-12), 74.7 (C-4), 68.0 (C-20), 66.8 (C-13), 57.3 (C-10), 44.4 (C-11), 40.0 (C-8), 38.4 (C-5), 37.2 (C-14), 35.4 (C-2″), 27.5 (C-15), 24.1 (C-17), 20.9 (C-2′), 19.0 (C-3″), 19.0 (C-4″), 17.3 (C-16), 14.8 (C-18), 10.2 (C-19).

phorbol 12,13-diacetate (**3**): ^1^H NMR (600 MHz, CD_3_OD): *δ*: 7.56 (1H, s, H-1), 5.63 (1H, d, *J* = 5.0 Hz, H-7), 5.43 (1H, d, *J* = 10.3 Hz, H-12), 3.97 (1H, d, *J* = 13.0 Hz, H-20α), 3.93 (1H, d, *J* = 13.0 Hz, H-20β), 3.18 (1H, m, H-8), 2.56 (1H, d, *J* = 19.0 Hz, H-5α), 2.51 (1H, d, *J* = 19.0 Hz, H-5β), 2.26 (1H, m, H-10), 2.07 (3H, s, H-2″), 1.82 (3H, s, H-2′), 1.81 (1H, m, H-11), 1.75 (3H, dd, *J* = 2.7, 1.1 Hz, H-19), 1.27 (3H, s, H-16), 1.24 (3H, s, H-17), 1.13 (1H, d, *J* = 5.3 Hz, H-14), 0.91 (3H, d, *J* = 6.5 Hz, H-18); ^13^C NMR (150 MHz, CD_3_OD): *δ*: 210.3 (C-3), 172.9 (C-1″), 171.2 (C-1′), 160.6 (C-1), 142.8 (C-6), 134.6 (C-2), 129.4 (C-7), 79.8 (C-9), 78.7 (C-12), 74.7 (C-4), 68.0 (C-20), 66.8 (C-13), 57.3 (C-10), 44.4 (C-11), 40.0 (C-8), 38.4 (C-5), 37.4 (C-14), 27.7 (C-15), 24.2 (C-17), 20.9 (C-2″), 17.3 (C-2′), 14.6 (C-16), 11.9 (C-18), 10.2(C-19).

12-*O*-acetylphorbol-13-(2″-methyl)butyrate (**4**): ^1^H NMR (600 MHz, CDCl_3_): *δ*: 7.56 (1H, br s, H-1), 5.62 (1H, d, *J* = 5.6 Hz, H-7), 5.42 (1H, d, *J =* 10.3 Hz, H-12), 3.97 (1H, d, *J =* 12.9 Hz, H-20α), 3.93 (1H, d, *J =* 12.9 Hz, H-20β), 3.30 (1H, m, H-8), 3.17 (1H, m, H-10), 2.55 (1H, d, *J =* 19.0 Hz, H-5α), 2.50 (1H, d, *J* = 19.0 Hz, H-5β), 2.41 (1H, m, H-2″), 2.24 (1H, m, H-11), 2.07 (3H, s, H-2′), 1.75 (3H, dd, *J* = 2.8, 1.3 Hz, H-19), 1.73 (1H, m, H-3″α), 1.50 (1H, m, H-3″β), 1.26 (3H, s, H-16), 1.23 (3H, s, H-17), 1.15 (3H, d, *J =* 7.0 Hz, H-5″), 1.08 (1H, d, *J =* 5.4 Hz, H-14), 0.96 (3H, t, *J =* 7.4 Hz, H-4″), 0.90 (3H, d, *J =* 6.5 Hz, H-18); ^13^C NMR (150 MHz, CDCl_3_): *δ*: 210.3 (C-3), 180.4 (C-1″), 172.7 (C-1′), 160.5 (C-1), 142.8 (C-6), 134.6 (C-2), 129.3 (C-7), 79.7 (C-9), 78.6 (C-12), 74.7 (C-4), 68.0 (C-20), 66.8 (C-13), 57.3 (C-10), 44.5 (C-11), 42.6 (C-14), 40.0 (C-8), 38.4 (C-5), 37.4 (C-2″), 27.6 (C-15), 27.4 (C-3″), 24.2 (C-17), 20.9 (C-2′), 17.4 (C-5″), 16.7 (C-16), 14.8 (C-4″), 12.0 (C-18), 10.2 (C-19).

12-*O*-tiglylphorbol-13-propionate (**5**): ^1^H NMR (600 MHz, CD_3_OD): *δ*: 7.55 (1H, s, H-1), 6.88 (1H, m, H-3′), 5.64 (1H, d, *J =* 4.7 Hz, H-7), 5.50 (1H, d, *J =* 10.4 Hz, H-12), 3.98 (1H, d, *J =* 13.0 Hz, H-20α), 3.94 (1H, d, *J =* 13.0 Hz, H-20β), 3.33 (1H, m, H-8), 3.18 (1H, m, H-10), 2.56 (1H, d, *J =* 19.0 Hz, H-5α), 2.51 (1H, d, *J =* 19.0 Hz, H-5β), 2.39 (2H, q, *J =* 15.0, 7.6 Hz, H-2″), 2.28 (1H, m, H-11), 1.84 (3H, s, H-5′), 1.83 (3H, d, *J =* 7.1 Hz, H-4′), 1.75 (3H, dd, *J =* 2.8, 1.3 Hz, H-19), 1.29 (3H, s, H-17), 1.21 (3H, s, H-16), 1.16 (1H, d, *J =* 5.2 Hz, H-14), 1.15 (3H, t, *J =* 7.5 Hz, H-3″), 0.88 (3H, d, *J =* 6.5 Hz, H-18); ^13^C NMR (150 MHz, CD_3_OD): *δ*:210.3 (C-3), 178.5 (C-1″), 169.5 (C-1′), 160.5 (C-1), 142.8 (C-6), 139.2 (C-3′), 134.6 (C-2), 129.6 (C-7), 129.3 (C-2′), 79.8 (C-9), 78.3 (C-12), 74.7 (C-4), 68.0 (C-20), 67.0 (C-13), 57.3 (C-10), 44.5(C-11), 40.0 (C-8), 38.4 (C-5), 37.2 (C-14), 28.6 (C-2″), 27.2 (C-15), 24.1 (C-16), 17.5 (C-17), 14.8 (C-4′), 14.5 (C-18), 12.3 (C-5′), 10.2 (C-19), 9.1 (C-3″).

12-*O*-acetyl-4α-deoxyphorbol-13-(2″-methyl)butyrate (**6**): ^1^H NMR (600 MHz, CD_3_OD): *δ*: 7.34 (1H, s, H-1), 5.47 (1H, d, *J =* 10.4 Hz, H-12), 5.13 (1H, s, H-7), 3.90 (1H, d, *J* = 14.0 Hz, H-20α), 3.86 (1H, d, *J =* 14.0 Hz, H-20β), 3.51 (1H, m, H-10), 3.35 (1H, m, H-5α), 2.75 (1H, m, H-4), 2.36 (1H, m, H-2″), 2.27 (1H, dd, *J* = 15.4, 4.6 Hz, H-5β), 2.11 (3H, s, H-2′), 1.98 (1H, br s, H-8), 1.92 (1H, s, H-3″α), 1.73 (3H, s, H-19), 1.71 (1H, m, H-11), 1.48 (1H, m, H-3″β), 1.28 (3H, s, H-17), 1.22 (3H, s, H-16), 1.11 (3H, d, *J* = 7.0 Hz, H-5″), 1.07 (3H, d, *J* = 6.4 Hz, H-18), 0.93 (3H, t, *J* = 7.4 Hz, H-4″), 0.82 (1H, d, *J* =5. 2 Hz, H-14); ^13^C NMR (150 MHz, CD_3_OD): *δ*: 214.3 (C-3), 180.3 (C-1″), 172.6 (C-1′), 159.1 (C-1), 144.1 (C-2), 137.8 (C-6), 124.7 (C-7), 79.6 (C-9), 77.3 (C-12), 69.1 (C-20), 66.5 (C-13), 50.3 (C-4), 48.4 (C-10), 44.3 (C-11), 42.6 (C-2″), 42.3 (C-8), 38.4 (C-14), 27.4 (C-3″), 27.2 (C-5), 27.1 (C-15), 24.5 (C-17), 20.9 (C-2′), 16.7 (C-16), 16.7 (C-5″), 12.2 (C-18), 12.0 (C-4″), 10.3 (C-19).

12-*O*-tiglyl-4-deoxy-4α-phorbol-13-acetate (**7**): ^1^H NMR (600 MHz, CD_3_OD): *δ*: 7.37 (1H, br s, H-1), 6.95 (1H, m, H-3′), 5.56 (1H, d, *J =* 10.6 Hz, H-12), 5.14 (1H, br s, H-7), 3.90 (1H, d, *J =* 13.9 Hz, H-20α), 3.86 (1H, d, *J =* 13.8 Hz, H-20β), 3.51 (1H, m, H-10), 3.35 (1H, m, H-5α), 2.75 (1H, m, H-4), 2.27 (1H, dd, *J =* 15.3, 4.7 Hz, H-5β), 2.04 (3H, s, H-2″), 2.00 (1H, m, H-8), 1.98 (1H, m, H-11), 1.89 (3H, s, H-5′), 1.86 (3H, d, *J =* 7.1 Hz, H-4′), 1.74 (3H, s, H-19), 1.31 (3H, s, H-17), 1.20 (3H, s, H-16), 1.05 (3H, d, *J =* 6.4 Hz, H-18), 0.92 (1H, d, *J =* 5.1 Hz, H-14); ^13^C NMR (150 MHz, CD_3_OD): *δ*: 214.4 (C-3), 175.2 (C-1″), 169.4 (C-1′), 159.1 (C-1), 144.0 (C-2), 139.4 (C-3′), 137.7 (C-6), 129.6 (C-2′), 124.7 (C-7), 79.6 (C-9), 77.1 (C-12), 69.0 (C-20), 66.8 (C-13), 50.3 (C-4), 48.4 (C-10), 44.3 (C-11), 42.3 (C-8), 38.1 (C-14), 27.2 (C-5), 26.6 (C-15), 24.4 (C-17), 21.0 (C-2″), 16.8 (C-16), 14.5 (C-4′), 12.3 (C-18), 12.2 (C-5′), 10.3 (C-19).

4α-deoxyphorbol 12-acetate-13-isobutyrate (**8**): ^1^H NMR (600 MHz, CD_3_OD): *δ*: 7.34 (1H, s, H-1), 5.45 (1H, d, *J* = 10.5 Hz, H-12), 5.13 (1H, s, H-7), 3.87 (2H, m, H-20), 3.50 (1H, m, H-10), 3.35 (1H, m, H-5a), 2.54 (1H, m, H-4), 2.26 (1H, d, *J* = 15.3 Hz, H-5b), 2.11 (3H, s, H-2′), 1.98 (1H, m, H-2″), 1.92 (1H, m, H-8), 1.74 (3H, m, H-19), 1.72 (1H, m, H-11), 1.28 (3H, s, H-17), 1.21 (3H, s, H-16), 1.15 (3H, d, *J* = 7.0 Hz, H-3″), 1.13 (3H, d, *J* = 7.0 Hz, H-4″), 1.07 (3H, d, *J* = 6.4 Hz, H-18), 0.84 (1H, d, *J* = 5.2 Hz, H-14); ^13^C NMR (150 MHz, CD_3_OD): *δ*: 214.4 (C-3), 180.6 (C-1″), 172.7 (C-1′), 159.1 (C-1), 144.1 (C-6), 137.8 (C-2), 124.8 (C-7), 79.6 (C-9), 77.1 (C-12), 69.1 (C-20), 66.5 (C-13), 49.6 (C-10), 48.3 (C-4), 44.2 (C-11), 42.2 (C-8), 38.3 (C-14), 35.4 (C-2″), 27.1 (C-5), 27.0 (C-15), 24.5 (C-16), 20.9 (C-2′), 19.0 (C-3″), 18.9 (C-4″), 16.6 (C-17), 12.2 (C-18), 10.2 (C-19).

12-*O*-acetyl-5,6-didehydro-7-oxophorbol-13-yl-2-methylpropanoate (**9**): ^1^H NMR (600 MHz, CD_3_OD): *δ*: 7.61 (1H, br s, H-1), 6.95 (1H, s, H-5), 5.41 (1H, d, *J =* 10.4 Hz, H-12), 4.26 (2H, m, H-20), 3.82 (1H, d, *J =* 5.5 Hz, H-8), 3.11 (1H, t, *J =* 2.6 Hz, H-10), 2.61 (1H, m, H-2″), 2.30 (1H, m, H-11), 2.08 (3H, s, H-2′), 1.81 (3H, d, *J =* 1.5 Hz, H-19), 1.76 (1H, d, *J =* 5.5 Hz, H-14), 1.22 (3H, s, H-17), 1.20 (3H, s, H-16), 1.19 (3H, d, *J* = 7.0 Hz, H-4″), 1.18 (3H, d, *J =* 7.0 Hz, H-3″), 0.95 (3H, d, *J =* 6.5 Hz, H-18); ^13^C NMR (150 MHz, CD_3_OD): *δ*: 205.7 (C-3), 202.1 (C-7), 180.8 (C-1″), 172.7 (C-1′), 158.9 (C-1), 149.7 (C-6), 138.3 (C-5), 136.8 (C-2), 77.9 (C-12), 77.2 (C-9), 74.3 (C-4), 66.9 (C-13), 62.4 (C-20), 60.3 (C-10), 55.7 (C-8), 46.0 (C-11), 35.4 (C-2″), 31.0 (C-14), 27.1 (C-15), 23.9 (C-16), 20.8 (C-2′), 19.0 (C-4″), 19.0 (C-3″), 17.1 (C-17), 14.8 (C-18), 10.4 (C-19).

12-*O*-acetyl-5,6-didehydro-7-oxophorbol-13-yl-2-methylbutanoate (**10**): ^1^H NMR (600 MHz, CD_3_OD): *δ*: 7.61 (1H, br s, H-1), 6.95 (1H, s, H-5), 5.43 (1H, d, *J =* 10.3 Hz, H-12), 4.28 (1H, d, *J =* 14.6 Hz, H-20α), 4.25 (1H, d, *J* = 14.6 Hz, H-20β), 3.83 (1H, d, *J =* 5.5 Hz, H-8), 3.12 (1H, m, *J* = 2.6 Hz, H-10), 2.43 (1H, m, H-2″), 2.32 (1H, m, H-11), 2.08 (3H, s, H-2′), 1.81 (3H, dd, *J =* 2.8, 1.3 Hz, H-19), 1.75 (1H, d, *J =* 5.5 Hz, H-14), 1.73 (1H, m, H-3″α), 1.50 (1H, m, H-3″β), 1.23 (3H, s, H-17), 1.20 (3H, s, H-16), 1.17 (3H, d, *J =* 7.1 Hz, H-5″), 0.97 (3H, t, *J =* 7.5 Hz, H-4″), 0.95 (3H, d, *J =* 6.5 Hz, H-18); ^13^C NMR (150 MHz, CD_3_OD): *δ*: 205.7 (C-3), 202.1 (C-7), 180.5 (C-1″), 172.7 (C-1′), 158.9 (C-1), 149.7 (C-6), 138.3 (C-5), 136.8 (C-2), 78.1 (C-12), 77.2 (C-9), 74.3 (C-4), 66.9 (C-13), 62.4 (C-20), 60.3 (C-10), 55.7 (C-8), 46.0 (C-11), 42.6 (C-2″), 31.1 (C-14), 27.4 (C-3″), 27.2 (C-15), 23.9 (C-16), 20.8 (C-2′), 17.2 (C-17), 16.7 (C-5″), 14.9 (C-18), 12.0 (C-4″), 10.4 (C-19).

### 3.4. ECD Calculation

Conformational analyses were carried out via random searching in Sybyl-X 2.0 using the MMFF94S force field with an energy cutoff of 5 kcal/mol. The results revealed the nine lowest energy conformers. Subsequently, geometry optimizations and frequency analyses were implemented at the B3LYP-D3(BJ)/6-31G* level in CPCM methanol using ORCA5.0.1 [32]. All conformers used for property calculations in this work were characterized to be at a stable point on a potential energy surface (PES) with no imaginary frequencies. The excitation energies, oscillator strengths, and rotational strengths (velocity) of the first 60 excited states were calculated using the TD-DFT methodology at the PBE0/def2-TZVP level in CPCM methanol using ORCA5.0.1 [32]. The ECD spectra were simulated by the overlapping Gaussian function (half the bandwidth at 1/e peak height, sigma = 0.30 for all) [33]. Gibbs free energies for conformers were determined using thermal correction at the B3LYP-D3(BJ)/6-31G* level and electronic energies evaluated at the wB97M-V/def2-TZVP level in CPCM methanol using ORCA5.0.1 [32]. To obtain the final spectra, the simulated spectra of the conformers were averaged according to the Boltzmann distribution theory and their relative Gibbs free energy (∆G). By comparing the experiment spectra with the calculated model molecules, the absolute configuration of the only chiral center was determined.

The ECD calculation results of compound **1** are available in the Supplementary Materials.

### 3.5. Killing of Schistosomula Test In Vitro

Oncomelania snails confirmed positive for schistosomiasis infection were provided by the Hubei Provincial Institute of Schistosomiasis Control. We left the snails in chlorinated water for 2 h at room temperature, and then collected the cercariae on the water surface into a 15 mL plastic centrifuge tube. Afterwards, the cercaria of Schistosoma collected were centrifuged and washed with pre-cooled medium at 4 °C twice. Every time of wash lasted for 5 min. Finally, the supernatant was discarded and the cercariae were collected. Schistosoma cercariae was added into a culture medium (containing 10% fetal bovine serum, 90% RPMI1640, 200 U/mL penicillin, 200 µg/mL streptomycin, 2.5 µg/mL amphotericin B and 10 mM 4-hydroxyethyl piperazine ethanesulfonic acid solution) and then submitted to inhaled syringe passes back and forth for certain times to shed its tail. After resuspension, it became schistosomula, and the tail breakage rate was more than 99%, as indicated by microscopic examination. After centrifuging, the severed tail was removed from the supernatant and schistosomula was collected. Schistosoma cercariae was divided into a blank control group, DMSO control group, praziquantel group and different concentrations of diterpenoid compounds group. During the experiment, samples **1**, **2**, **3**, **4**, **5**, **6**, **7**, **8**, **9**, and **10** were taken and dissolved with a little DMSO. Culture medium and distilled water were added for ultrasound suspension, and the concentrations of 8.50, 17.00, and 34.00 μg/mL in vitro test culture medium were prepared. Meanwhile, after dissolving praziquantel with a little DMSO, we added culture medium and distilled water for ultrasound suspension and prepared them into concentrations of 30.00 μg/mL in vitro test culture medium. 

A measure of 1 mL culture medium was added to a centrifuge tube containing schistosomula, drawn well, and dripped onto a 6-well plate with 40 schistosomula per well. According to the above groups, 2 mL of drug-containing culture medium was added to each group, and culture medium was used for the blank control group. The 6-well plate was incubated in a 37 °C CO_2_ incubator, and the changes and survival status of the worms were observed under a microscope at 24, 48, and 72 h. Finally, the survival rate of the worms was calculated.

### 3.6. Cell Culture

Human hepatic stellate cell LX2 is a non-parenchymal cell type, and LX2 cells usually serve as an in vitro model for liver fibrosis study, which helps to understand the occurrence and development of liver fibrosis. LX2 cells are a living cell line derived from the human liver, characterized by astrocytes, phenotype stability, expressing liver-specific markers, and so on. The features of LX2 cells make them an important tool for studying the mechanism of liver fibrosis, as well as screening for new compounds. LX-2 were provided by Abiowell Biotechnology Co., Ltd. (Changsha, China). The cell cryopreservation tubes in liquid nitrogen were transferred into a 37 °C water bath for thawing, and then the supernatant was centrifuged and discarded, adding 1 mL of complete medium (10% fetal bovine serum, 100 U/mL penicillin, 0.1 mg/mL streptomycin) to resuspend the cells. We transferred the cell suspension into a culture flask and added 4 mL of complete medium, gently stirred, and observed the cell morphology under a microscope. The cells were incubated at 37 °C in a humidified atmosphere containing 5% CO_2_. When the cells covered 70–80% of the culture dish, they could be passed on. The cells were at a ratio of 1:3 and gently blow-dried to evenly distribute them, and cells could be frozen and stored in a liquid nitrogen tank when the cell growth conditions were good. 

### 3.7. Cytotoxicity Testing

The cells were divided into a blank control group, compound **2** treatment group (10.00, 20.00, 40.00, 80.00, 160.0 μM), compound **4** treatment group (20.00, 40.00, 80.00, 160.0, 320.0 μM), and compound **10** treatment group (10.00, 20.00, 40.00, 80.00, 160.0 μM). Each group had 6 wells, and the experiment was repeated 3 times. The CCK-8 method (https://www.abiowell.com/fuzhushiji/1271.htm, accessed on 9 January 2023) was used to detect cell proliferation. LX-2 cells were collected in the logarithmic growth stage, adjusting the cell concentration to 5 × 10^3^ mL. Cells were seeded in 100 μL per well in 96-well plates. After overnight cell culture, the treatment group received specific drug stimulation, while the control group did not receive treatment. First, 100 µL of prepared cell culture medium was added to each well after washing twice with PBS, and then 10 µL of CCK-8 solution was added to each well. After 4 h of incubation in a cell incubator, the absorbance of each well at 450 nm was measured using a multifunctional enzyme-linked immunosorbent assay. Using SPSS26.0 statistical software, the cell growth inhibition rates of different concentrations of drugs on influenza viruses were calculated, and probit regression analysis was used to calculate the half inhibitory concentration (IC_50_) of the drug.

Cell growth inhibition rate = (normal group OD value − sample group OD value/normal group OD value) × 100%

### 3.8. Establishing Cell Models

Normal LX-2 cells were seeded into a 96-well plate with 1 mL per well, incubated overnight to allow the cells to adhere to the wall, and changed to serum-free medium and starved for 12 h to synchronize the cell growth cycle. In addition to the blank control group, 5 ng/mL TGF-β1 was added to each treatment group to stimulate cells for 12 h to activate them. TGF-β1 is a recognized factor that promotes the formation of liver fibrosis. During the process of liver fibrosis, TGF-β1 induces stromal cell proliferation and transformation into fibroblasts, increases the synthesis of collagen and other matrix components, and aggravates the deposition of cellulose, collagen, and elastin, thereby causing liver fibrosis.

### 3.9. The Extraction of Total RNA and Protein of the Cells 

The LX-2 cells were divided into a negative control group; TGF-β1 model group; the low-, middle-, and high-dose groups of compound **2** (0.50 µM, 1.00 µM, 2.00 µM); the low-, middle-, and high-dose groups of compound **4** (3.00 µM, 6.00 µM, 12.00 µM); the low-, middle-, and high-dose groups of compound **10** (1.25 µM, 2.50 µM, 5.00 µM); colchicine control group (2.5 μg/mL); and SB431542 group (10 μmol/L). In addition to the negative control group, all other groups were treated with TGF-β1. After 2 h of adsorption, the supernatant was discarded, and then cleaning was performed twice with PBS, followed by the addition of different concentrations of the compounds mentioned above. The total RNA and total protein of the cells were extracted after 48 h of exposure. The mRNA and protein expression of COL-I, COL-III, α-SMA, TGF-β1, TGFβRI, TGFβRII, Smad2, and Smad3 were detected by RT-qPCR and Western blot assay, respectively.

### 3.10. ELISA Detection

The contents of COL-I, COL-III, α-SMA, TGF-β1 were examined by ELISA assay. The reagent kit and sample to be tested were taken out of the refrigerator 20 min in advance to increase to room temperature, and then the diluted liquid of the sample was added to the well plate and incubated at 37 °C for 90 min. Next, we dried the liquid in the orifice plate and added 1× incubated biotinylated antibody working solution under the same conditions for 1 h, and then dried the liquid in the well plate and added 350 μL 1× washed liquid to each well, shaking and mixing well for 1 min, followed by shaking to dry and patting dry with thick absorbent paper. Next, 100 μL 1× enzyme conjugate working solution was added to each well, which was incubated in the dark at 37 °C for 30 min after coating with a sealing film. After washing the plate 3 times, 90% of the substrate was added to each well. The plate was incubated in the dark at 37 °C for 15 min; finally, 50 μL of the termination solution was added to each well to terminate the reaction, and an enzyme-linked immunosorbent assay was used to detect the optical density (OD value) at a wavelength of 450 nm for different pores. Then, the OD value of the blank control well was subtracted from the OD value of the detection well as the calibration value, and the standard curve was drawn with the concentration as the horizontal axis and the standard OD value as the vertical axis. The OD value of the test sample was substituted into the standard curve to obtain the detection concentration of the test sample.

### 3.11. RT-PCR Detection 

The mRNA levels of COL-I, COL-III, α-SMA, TGF-β1, TGFβRI, TGFβRII, Smad2, and Smad3 were detected by RT-qPCR assay. Total RNA from cells were extracted using the Trizol method, and its concentration was measured using a UV spectrophotometer. Its absorbance values at 260 and 280 nm were calculated to determine the concentration and purity. Then, cell RNA agarose gel electrophoresis was performed, and the gel imaging system was used for observation and photography. Transcription of cDNA was reversed using cell total RNA as a template. The specific reaction system, operational steps, and reverse transcription conditions are outlined in Table 4. The reaction system was mixed well, and a vortex oscillator was used to centrifuge at 2000 rpm for 10 s, allowing the solution on the tube wall to be collected at the bottom of the tube. The tube was incubated in a 42 °C water bath for 60 min and then in an 85 °C water bath for 5 min. After the reaction was completed, centrifugation was performed at 3000 rpm for 10 s, and then the tube was placed on ice for cooling. Reverse transcripts can be directly used for fluorescence quantitative PCR reactions. The sequences of the target genes were searched on NCBI, and the primer sequences of COL-I, COL-III, α-SMA, TGF-β1, TGFβRI, TGFβRII, Smad2, and Smad3 were, respectively, designed using primer5 software; the primer sequences for each gene are shown in Table 5. Briefly, 30 μL of reaction volume contained 15 μL of SYBR Green PCR Master Mix, 1 μL of Primer R (10 μM), 1 μL of Primer F (10 μM), 11 μL of diethyl pyrocarbonate (DEPC)-treated water and 2 μL of template.

The optimum conditions for the PCR amplification of the cDNA were established by following the manufacturer’s instructions. The PCR cycle conditions were 95 °C, pre-denaturation for 10 min, then 95 °C for 15 s and 60 °C for 30 s, repeated for 40 cycles. The dissolution curves were determined at 60–95 °C. The data were analyzed using StepOne software (version 2.3) (Applied Biosystems, Waltham, MA, USA), and the cycle numbers at the linear amplification threshold values (Ct) for the endogenous dog GAPDH gene and the target genes were recorded. Relative gene expression (target gene expression normalized to the expression of the endogenous GAPDH gene) was calculated using the comparative Ct method (2^−∆∆Ct^). The experiments and analysis were conducted independently three times.

### 3.12. Western Blotting Detection

The total proteins of cells were extracted and lysed in each test group, and the concentration of sample protein in each group was measured with a BCA protein quantitative kit. We prepared sodium dodecyl sulfate polyacrylamide gel for electrophoresis, transferred the semi-dry electric membrane converter to NC membrane, and added the corresponding TGF-β1 primary antibody (diluted with 1:2000) after sealing skimmed milk. The membrane and primary antibody were incubated in a refrigerator at 4 °C overnight. The membrane and secondary antibody were incubated at room temperature for 90 min. After incubation, ECL chemiluminescence solution was used for color development, and X-ray film was exposed for several seconds to several minutes. The results were observed through development and fixation scanning. The grayscale of the target protein bands in the scanned image was analyzed using Image-ProPlus software (version 6.0). The gray ratio of each target band and β-actin is the relative expression of the target protein. The protein expressions of COL-I, COL-III, α-SMA, TGF-β1, TGFβRI, TGFβRII, Smad2, and Smad3 were detected by Western blot assay.

### 3.13. Immunohistochemical Detection

The expressions of COL-I, COL-III, α-SMA, TGF-β1, TGFβRI, TGFβRII, Smad2, and Smad3 were measured by immunohistochemical staining assay. The climbing film was fixed with 4% paraformaldehyde for 30 min and was then flushed with PBS three times for 5 min each time, and it was then added to 0.3% tramadol and allowed to penetrate for 30 min at 37 °C. The climbing film was added to 3% H_2_O_2_ at room temperature for 10 min to inactivate endogenous enzymes. The climbing film was incubated with primary antibodies (COL-I, COL-III, α-SMA, TGF-β1, TGFβRI, TGFβRII, Smad2, Smad3) overnight at 4 °C. The climbing film was incubated with secondary antibody at 37 °C for 30 min and was rinsed with PBS three times for 5 min each time. The climbing film was incubated with DAB working solution for 1 min and was washed with distilled water. The climbing film was stained with hematoxylin for 5–10 min and was rinsed with distilled water and then returned to blue with PBS. The climbing film was dehydrated with all levels of alcohol (60–100%) for 5 min per level. After removal, the climbing film was placed in xylene for 10 min and was sealed with neutral gum for observation under a microscope.

### 3.14. Statistical Analysis

The data were processed using SPSS26.0. Measurement data (x ± s) were represented, significance comparison was performed using analysis of variance, inter-group comparison was performed using one-way ANOVE, and inter-group comparison was performed using LSD multiple analysis. The difference was statistically significant with *p* < 0.05.

## 4. Conclusions

At present, chemotherapy is the main means of treating schistosomiasis, and praziquantel is the preferred treatment drug [13,14,15]. However, praziquantel has some mild side effects such as headache, nausea, anorexia, and reports of drug resistance [16,17]. In this research, we found that all diterpenoids isolated from the leaves of *C. tiglium* have a stronger insecticidal effect on schistosomula, and that compounds **2**, **4**, and **10** have good anti-liver-fibrosis effects. As far as we know, this is the first report of the compounds from *C. tiglium* showing activities of schistosomula killing and anti-liver-fibrosis. These data suggest that diterpenoids from *C. tiglium* may serve as potential schistosomula-killing and anti-liver-fibrosis agents in the future.

In the pathogenesis of schistosomiasis, the biggest damage is secondary fibrosis caused by Schistosoma cercariae eggs deposited in the liver [8,9]. TGF-β_1_ is currently recognized as a pro-fibrotic factor and has the effects of activating hepatic stellate cells, promoting collagen synthesis, and ultimately leading to liver fibrosis [10]. In the process of liver fibrosis disease, TGF-β1 promotes the production of extracellular matrix, reduces the degradation of COL-I and COL-III, and aggravates liver fibrosis. TGF-β ligands bind with TGFβRII on the cell surface, activate the serine/threonine kinase region of TGFβRI, forming heterotrimer, TGFβRI phosphorylates, and activate Smad2 and Smad3 to form an active transcription complex into the nucleus, and the signal is transferred from the cytoplasm to the nucleus to regulate gene transcription. The results in this research show that compounds **2**, **4**, and **10** have good anti-liver-fibrosis effects and that compound **2** can regulate the expression of TGF-β/Smad-pathway-related proteins. Our experimental results suggest that compound **2** plays an anti-liver-fibrosis role in regulating the TGF-β1/Smad signaling pathway.

In conclusion, one new tigliane-type diterpene and nine known analogues were isolated from the leaves of *C. tiglium*. We first demonstrated, in vitro, the potential schistosomula-killing and anti-liver-fibrosis effects of diterpenoids from *C. tiglium*. Our findings offer a potential new schistosomula-killing and anti-liver-fibrosis agent for medicinal applications. However, the safe usage and application of these diterpenoids should be further investigated in animal models.

## Figures and Tables

**Table 1:** ^1^H and ^13^C NMR data for **1** (600, 150 MHz, CD_3_OD, TMS, *δ* ppm).

Position	*δ*_C_	*δ*_H_ (*J* in Hz)
1	159.4, CH	7.60, s
2	133.1, C	
3	208.8, C	
4	73.1, C	
5	37.6, CH_2_	2.58, d (18.4) 2.42, d (18.4)
6	136.2, C	
7	132.5, CH	5.72, dd (5.5, 1.7)
8	39.0, CH	3.31, t (5.6)
9	78.2, C	
10	56.2, CH	3.11, t (2.5)
11	44.9, CH	2.04, m
12	76.2, CH	3.89, d (9.8)
13	67.5, C	
14	35.2, CH	1.05, d (5.4)
15	25.8, C	
16	16.0, CH_3_	1.26, s
17	22.9, CH_3_	1.27, s
18	14.1, CH_3_	1.09, d (6.5)
19	8.8, CH_3_	1.77, dd (2.7, 1.2)
20	69.2, CH_2_	4.50, s
1′	171.2, C	
2′	19.4, CH_3_	2.04, s
1″	179.8, C	
2″	41.1, CH	2.47, m
3″	26.2, CH_2_	1.74, m 1.53, m
4″	10.6, CH_3_	0.98, t (7.4)
5″	15.5, CH_3_	1.20, d (7.0)

**Table 2:** The effect of compounds on schistosomula killing in vitro.

Group	Concentration/(μg/mL)	Number of Schistosoma Cercariae per Hole/Piece	Survival Rate of Schistosomula/%		
			24	48	72
Blank control group	---	40	0.92 ± 0.04	0.86 ± 0.03	0.75 ± 0.04
Praziquantel	30.00	40	0.43 ± 0.08 *	0.28 ± 0.05 *	0 *
DMSO	---	40	0.89 ± 0.07	0.83 ± 0.04	0.70 ± 0.04
	8.50	40	0.39 ± 0.03 *	0.22 ± 0.03 *	0 *
1	17.00	40	0.38 ± 0.07 *	0.21 ± 0.05 *	0 *
	34.00	40	0.22 ± 0.01 *#	0.14 ± 0.02 *#	0 *
	8.50	40	0.35 ± 0.02 *	0.23 ± 0.01 *	0 *
2	17.00	40	0.28 ± 0.06 *#	0.20 ± 0.05 *	0 *
	34.00	40	0.20 ± 0.05 *#	0.12 ± 0.04 *#	0 *
	8.50	40	0.39 ± 0.03 *	0.23 ± 0.03 *	0 *
3	17.00	40	0.42 ± 0.04 *	0.22 ± 0.05 *	0 *
	34.00	40	0.24 ± 0.01 *#	0.12 ± 0.02 *#	0 *
	8.50	40	0.34 ± 0.02 *	0.23 ± 0.01 *	0 *
4	17.00	40	0.29 ± 0.06 *#	0.20 ± 0.05 *	0 *
	34.00	40	0.21 ± 0.05 *#	0.16 ± 0.04 *#	0 *
	8.50	40	0.36 ± 0.04 *	0.22 ± 0.03 *	0 *
5	17.00	40	0.43 ± 0.04 *	0.19 ± 0.04 *	0 *
	34.00	40	0.22 ± 0.04 *#	0.14 ± 0.03 *#	0 *
	8.50	40	0.39 ± 0.01 *	0.23 ± 0.01 *	0 *
6	17.00	40	0.44 ± 0.09 *	0.20 ± 0.05 *	0 *
	34.00	40	0.22 ± 0.04 *#	0.16 ± 0.04 *#	0 *
	8.50	40	0.39 ± 0.02 *	0.22 ± 0.01 *	0 *
7	17.00	40	0.47 ± 0.04 *	0.22 ± 0.08 *	0 *
	34.00	40	0.22 ± 0.03 *#	0.11 ± 0.03 *#	0 *
	8.50	40	0.39 ± 0.01 *	0.23 ± 0.01 *	0 *
8	17.00	40	0.38 ± 0.05 *	0.20 ± 0.05 *	0 *
	34.00	40	0.24 ± 0.01 *#	0.12 ± 0.01 *#	0 *
	8.50	40	0.44 ± 0.03 *	0.22 ± 0.01 *	0 *
9	17.00	40	0.47 ± 0.09 *	0.20 ± 0.03 *	0 *
	34.00	40	0.21 ± 0.01 *#	0.13 ± 0.02 *#	0 *
	8.50	40	0.39 ± 0.02 *	0.23 ± 0.01 *	0 *
10	17.00	40	0.34 ± 0.06 *	0.23 ± 0.05 *	0 *
	34.00	40	0.20 ± 0.05 *#	0.12 ± 0.04 *#	0 *

Note: Compared with the blank control group, * *p* < 0.05; compared with praziquantel, # *p* < 0.05.

**Table 3:** The cytotoxicity of compounds **2**, **4**, and **10** on LX-2 cells.

Compounds	Concentration (μM)	Inhibition Rate (%)	IC_50_ (μM)	TC_0_ (μM)
	10	4.15 ± 4.30		
	20	9.61 ± 1.91		
Compound **2**	40	21.44 ± 3.98	103.89	2.14
	80	37.37 ± 5.20		
	160	47.41 ± 2.00		
	20	4.96 ± 1.18		
	40	14.98 ± 1.63		
Compound **4**	80	20.83 ± 2.24	123.29	5.17
	160	44.47 ± 1.84		
	320	53.81 ± 1.46		
	20	9.47 ± 1.88		
	40	17.82 ± 1.72		
Compound **10**	80	28.90 ± 1.06	315.01	11.80
	160	46.39 ± 2.92		
	320	58.32 ± 3.22		

**Table 4:** Reverse transcription system.

Reagent	20 µL Reaction System	Final Concentration
dNTP Mix, 2.5 mM Each	4 µL	500 µM Each
Primer Mix	2 µL	
RNA Template	7 µL	50 pg–5 µg
5 × RT Buffer	4 µL	1×
DTT, 0.1 M	2 µL	10 mM
HiFiScript, 200 U/µL	1 µL	

**Table 5:** Primer sequence of COL-I, COL-III, α-SMA, TGF-β1, TGFβRI, TGFβRII, Smad2, and Smad3.

Primer Name	Forward	Revers
GAPDH	ACAGCCTCAAGATCATCAGC	GGTCATGAGTCCTTCCACGAT
TGF-β1	AGCAACAATTCCTGGCGATACCTC	CAATTTCCCCTCCACGGCTCA
COLI	GCAAGAACCCCGCCCGCACC	GCTCTCGCCGAACCAGACATGCC
COLIII	CGCCCTCCTAATGGTCAAGG	TTCTGAGGACCAGTAGGGCA
α-SMA	CCTGAGCGTTTTGATGCCTT	ACTTCAGCCGATAGTTTGTCT
TGFβR1	CCTCGAGATAGGCCGTTTGTA	ATGGTAAACCAGTAGTTGGAAGT
TGFβR2	CGTGAAGAACGACCTAACC	CCACCTGCCCACTGTTAG
SMAD2	TCCATCTTGCCATTCAC	TTCTTCCTCCCCATTCT
SMAD3	GCGTGCGGCTCTACTACAT	CTGGTAGACAGCCTCAAAGC

## Data Availability

Data are contained within this article.

## Supplementary Materials

The following supporting information can be downloaded at: https://www.mdpi.com/article/10.3390/molecules29020401/s1, Figure S1: The ^1^H NMR spectrum of compound **1**; Figure S2: The ^13^C NMR spectrum of compound **1**; Figure S3: The DEPT spectrum of compound **1**; Figure S4: The HSQC spectrum of compound **1**; Figure S5: The ^1^H-^1^H COSY spectrum of compound **1**; Figure S6: The HMBC spectrum of compound **1**; Figure S7: The ROESY spectrum of compound **1**; Figure S8: The HRESIMS spectrum of compound **1**; Table S1: The ECD calculation results of compound **1**.
