# Supplementary material for: Diterpenoids with Schistosomula-Killing and Anti-Fibrosis Activities In Vitro from the Leaves of Croton tiglium
Source: Molecules. 2024 Jan 13;29(2):401. doi: 10.3390/molecules29020401 (PMC10818740; doi:10.3390/molecules29020401)
Supplement: Supplementary file 1 [file molecules-29-00401-s001.zip › molecules-2766899-supplementary.pdf]

Supplementary data

Diterpenoids with Schistosomula-Killing and Anti-Fibrosis Activities In Vitro from the Leaves of *Croton tiglium*

Li Li [1,†], Biqing Zhao [1,†], Xiaoxiao Zheng [1], Zhaohui Liu [2], Huan Zou [2], Li Qin [1,*] and Xiaojiang Zhou [1,*]

[1] College of Pharmacy, Hunan University of Chinese Medicine, Changsha 410208, China; lili19950711@126.com (L.L.); qingerhn@126.com (B.Z.); xiao1320329479@163.com (X.Z.)
[2] Hengxiu Tang Pharmaceutical Co., Ltd., Changsha 410219, China; liuzhaohui@yfpharmacy.com (Z.L.); zouhuan@yfpharmacy.com (H.Z.)
* Correspondence: lqin1011@126.com (L.Q.); 003568@hnucm.edu.cn (X.Z.); Tel.: +86-731-88458238 (L.Q.); +86-731-88458234 (X.Z.); Fax: +86-731-88458227 (L.Q. & X.Z.)
† These authors contributed equally to this work.

List of Supporting Information

List of contents **Page**

Figure S1. The ^{1}H NMR spectrum of compound (**1**) in CD_3OD……….……………………………S3

Figure S2. The ^{13}C NMR spectrum of compound (**1**)CD_3OD…….….….….….….….….….….……S3

Figure S3. The DEPT spectrum of compound (**1**)CD_3OD……………………………………S4

Figure S4. The HSQC spectrum of compound (**1**) in CD_3OD….………….….……….……....…...S4

Figure S5. The ^{1}H-^{1}H COSY spectrum of compound (**1**) in CD_3OD….………….….………….….…S5

Figure S6. The HMBC spectrum of compound (**1**) in CD_3OD….………….….…………..………......S5

Figure S7. The ROESY spectrum of compound (**1**) in CD_3OD….………….….……..………..………S6

Figure S8. The HRESIMS spectrum of compound (**1**)..………….….….….…...….….….………S6

Table S1. The ECD calculation results of compound (**1**)……..……………………………S7

Figure S1. The ^{1}H NMR spectrum of compound (**1**) in CD_3OD

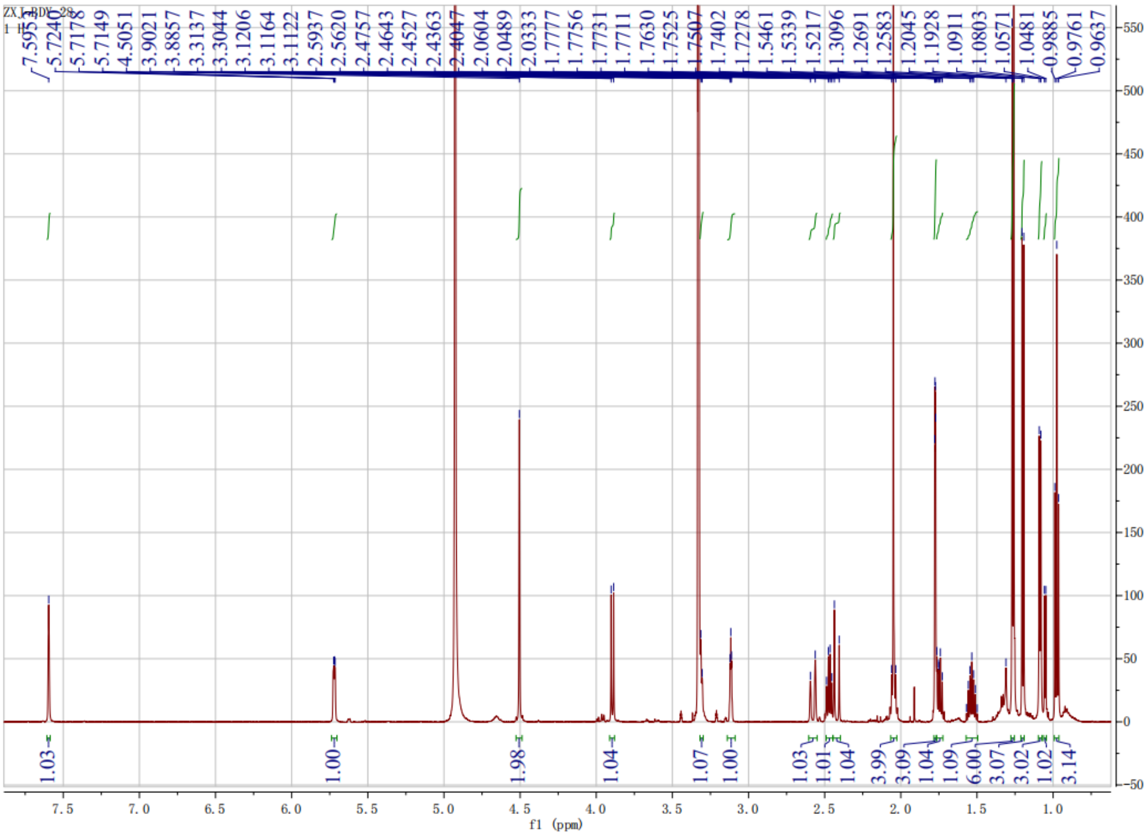

Figure S2. The ^{13}C NMR spectrum of compound (**1**) in CD_3OD

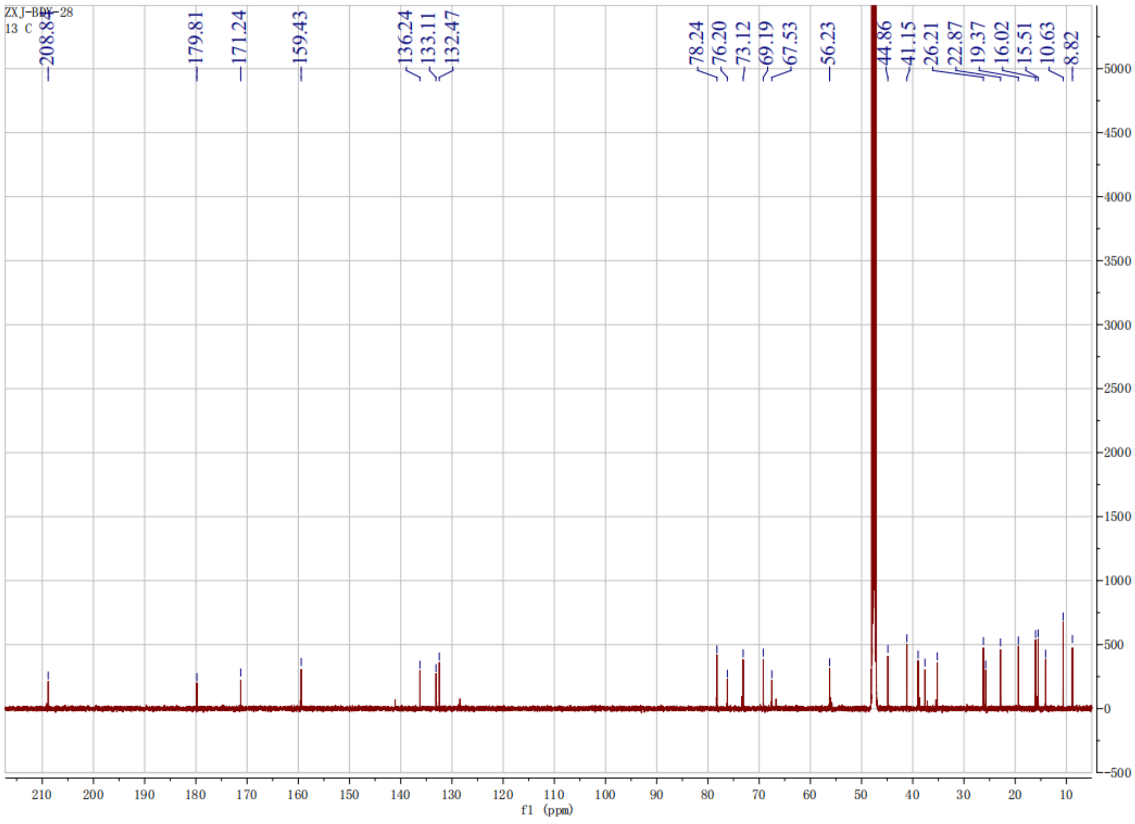

Figure S3. The DEPT spectrum of compound (**1**) in CD3OD

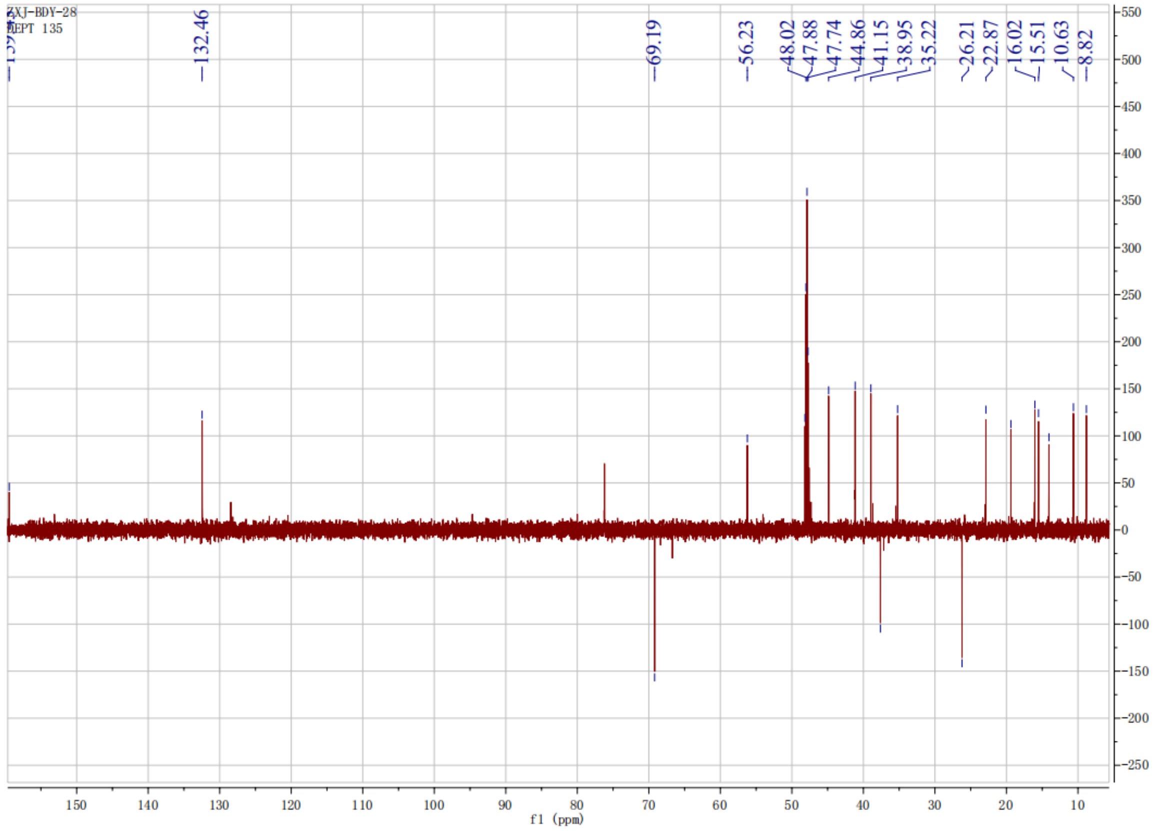

Figure S4. The HSQC spectrum of compound (**1**) in CD3OD

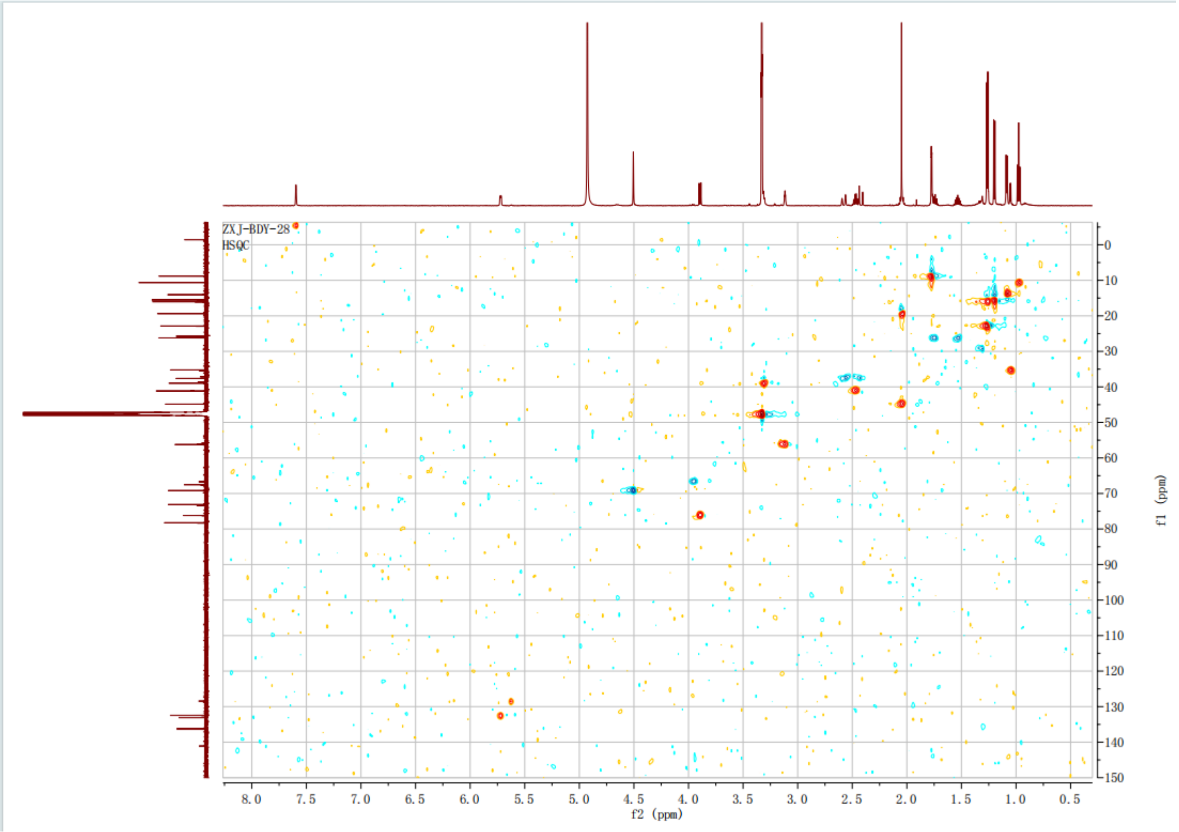

Figure S5. The ^{1}H-^{1}H COSY spectrum of compound (**1**) in CD_3OD

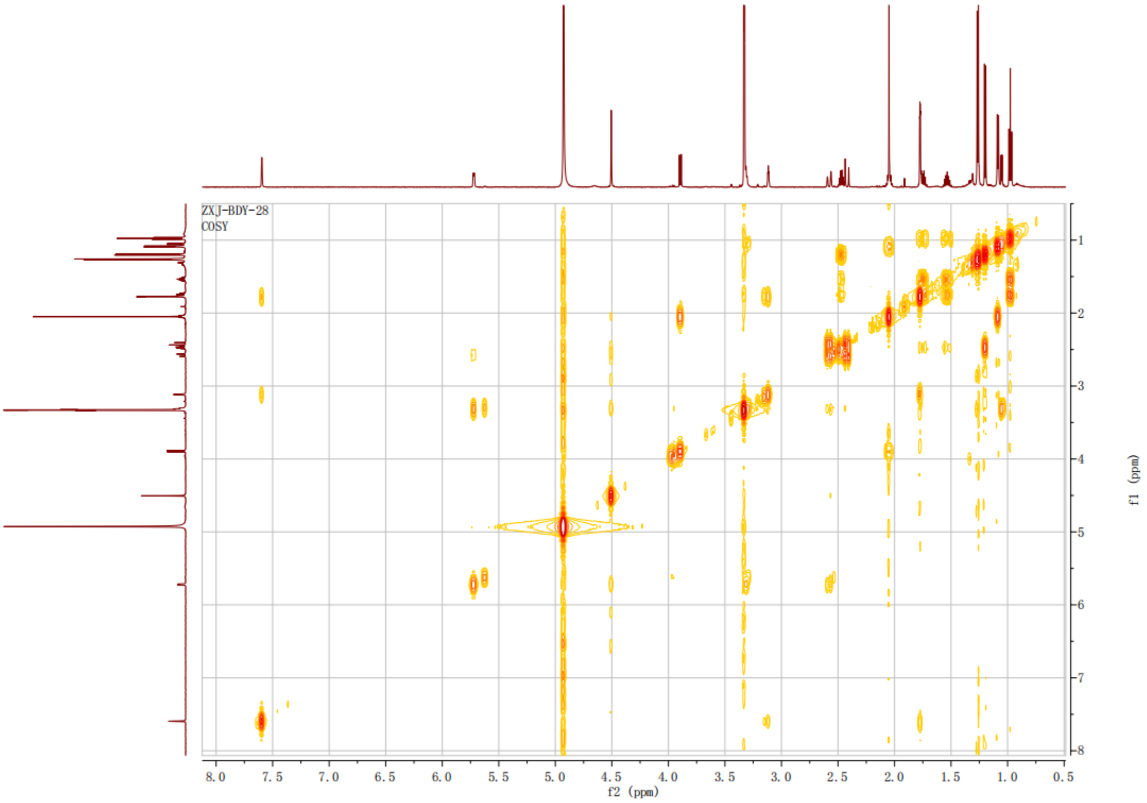

Figure S6. The HMBC spectrum of compound (**1**) in CD_3OD

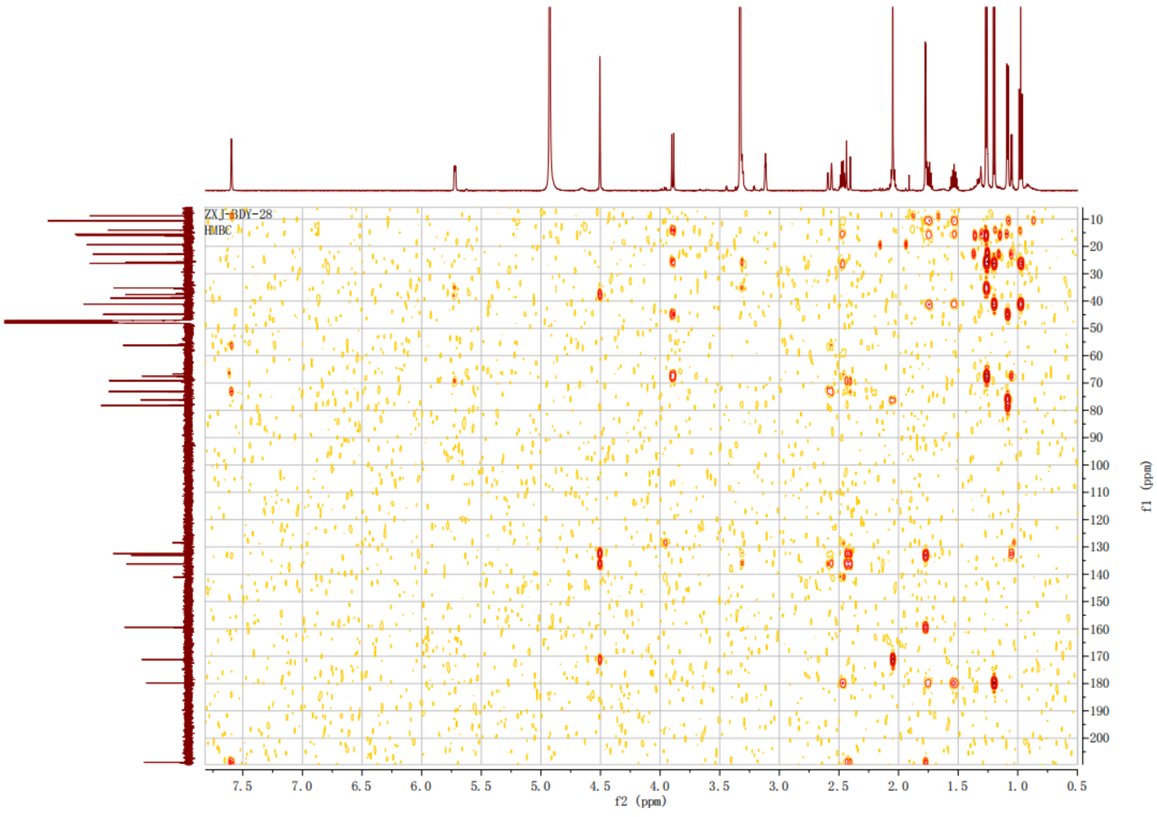

Figure S7. The ROESY spectrum of compound (**1**) in CD_3OD

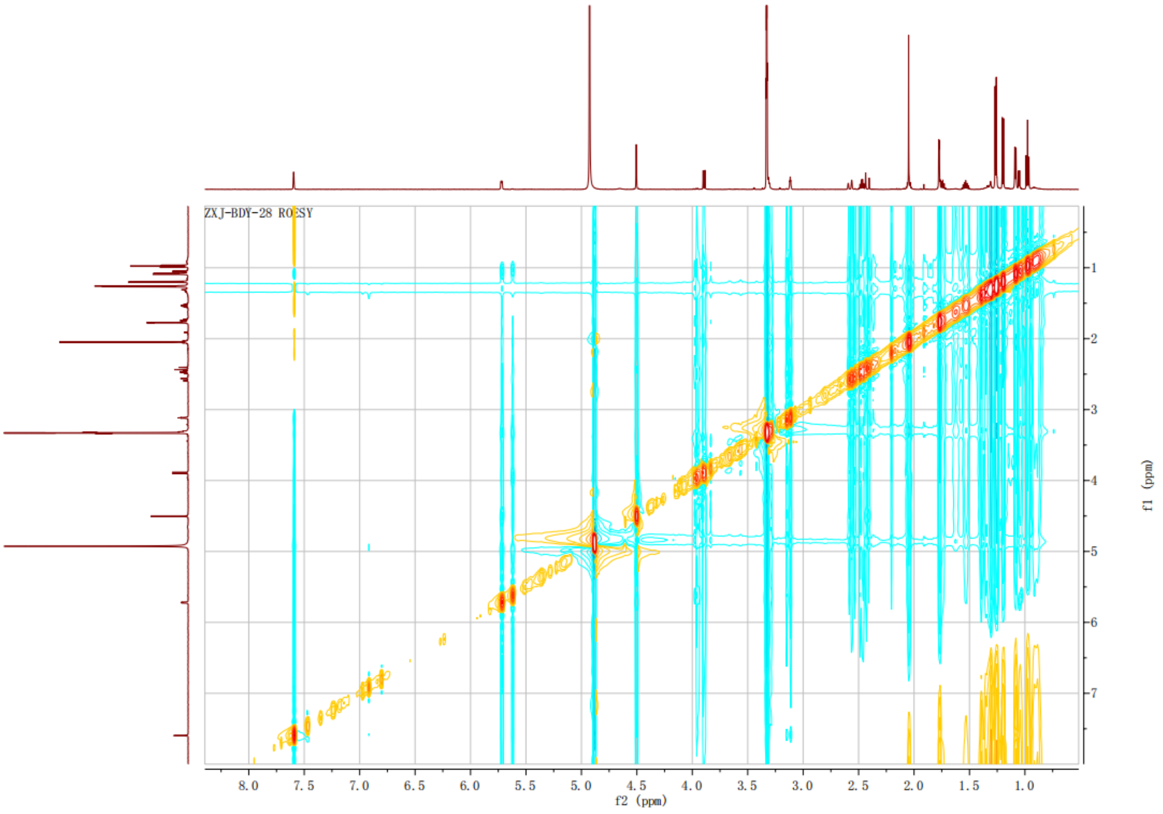

Figure S8. The HRESIMS spectrum of compound (**1**)

Mass Spectrum SmartFormula Report

**Analysis Info**		Acquisition Date	12/6/2022 4:40:57 PM
Analysis Name	D:\Data\MS\data\202212\lili_BDY-28_pos_1_01_14083.d		
Method	LC_Direct Infusion_pos_70-500mz.m	Operator	SCSIO
Sample Name	lili_BDY-28_pos	Instrument	maXis 255552.00029
Comment			

Acquisition Parameter

Source Type	ESI	Ion Polarity	Positive	Set Nebulizer	0.4 Bar
Focus	Active	Set Capillary	4500 V	Set Dry Heater	180 °C
Scan Begin	100 m/z	Set End Plate Offset	-500 V	Set Dry Gas	4.0 l/min
Scan End	1500 m/z	Set Charging Voltage	0 V	Set Divert Valve	Waste
		Set Corona	0 nA	Set APCI Heater	0 °C

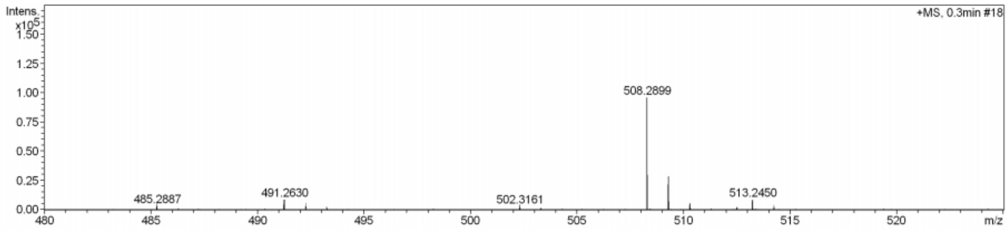

Meas. m/z	#	Ion Formula	Score	m/z	err [ppm]	err [mDa]	mSigma	rdb	e⁻ Conf	N-Rule
491.2630	1	C27H39O8	100.00	491.2639	1.9	0.9	28.6	8.5	even	ok
508.2899	1	C27H42NO8	100.00	508.2905	-1.2	-0.6	3.5	7.5	even	ok
513.2450	1	C27H38NaO8	100.00	513.2459	-1.7	-0.9	27.7	8.5	even	ok

lili_BDY-28_pos_1_01_14083.d
Bruker Compass DataAnalysis 4.1   printed: 12/6/2022 4:51:14 PM   by: SCSIO   Page 1 of 1

Table S1. The ECD calculation results of compound (**1**)

Wavelength	Experiment	Wavelength	Calculate a	Calculate b
400	-0.0488749	200	-37.49666667	37.49666667
399	0.165462	201	-37.93	37.93
398	-0.186818	202	-37.75666667	37.75666667
397	-0.0256567	203	-36.93666667	36.93666667
396	-0.03728	204	-35.64	35.64
395	0.0385857	205	-33.88333333	33.88333333
394	-0.033999	206	-31.57333333	31.57333333
393	0.0736322	207	-29.08	29.08
392	0.0909897	208	-26.32666667	26.32666667
391	0.0955913	209	-23.37	23.37
390	0.146887	210	-20.05333333	20.05333333
389	0.268319	211	-16.87333333	16.87333333
388	0.329287	212	-13.67	13.67
387	0.299917	213	-10.25333333	10.25333333
386	0.350415	214	-7.13	7.13
385	0.313146	215	-4.11	4.11
384	0.191211	216	-1.213333333	1.213333333
383	0.142523	217	1.713333333	-1.713333333
382	0.131831	218	4.266666667	-4.266666667
381	0.00260554	219	6.626666667	-6.626666667
380	-0.0501322	220	8.93	-8.93
379	0.0602837	221	10.86666667	-10.86666667
378	0.133611	222	12.58666667	-12.58666667
377	0.142876	223	14.1	-14.1
376	0.159963	224	15.49333333	-15.49333333
375	0.149487	225	16.58	-16.58
374	0.110348	226	17.47	-17.47
373	0.0621606	227	18.22	-18.22
372	-0.00706423	228	18.73666667	-18.73666667
371	-0.144532	229	19.09	-19.09
370	-0.21649	230	19.28666667	-19.28666667
369	-0.191244	231	19.34	-19.34
368	-0.210723	232	19.26	-19.26
367	-0.282398	233	19.06	-19.06
366	-0.268281	234	18.72333333	-18.72333333
365	-0.274768	235	18.31666667	-18.31666667
364	-0.211452	236	17.82333333	-17.82333333
363	-0.177976	237	17.25666667	-17.25666667
362	-0.184646	238	16.58	-16.58
361	-0.188737	239	15.89	-15.89
360	-0.342567	240	15.15333333	-15.15333333
359	-0.50398	241	14.32	-14.32

358	-0.665253	242	13.51	-13.51
357	-0.73118	243	12.67	-12.67
356	-0.694505	244	11.82	-11.82
355	-0.72569	245	10.88666667	-10.88666667
354	-0.702287	246	10.00666667	-10.00666667
353	-0.697127	247	9.123333333	-9.123333333
352	-0.678006	248	8.173333333	-8.173333333
351	-0.714113	249	7.29	-7.29
350	-0.842369	250	6.413333333	-6.413333333
349	-1.01044	251	5.543333333	-5.543333333
348	-1.04804	252	4.626666667	-4.626666667
347	-1.0547	253	3.78	-3.78
346	-0.979635	254	2.956666667	-2.956666667
345	-0.934562	255	2.09	-2.09
344	-0.952729	256	1.3	-1.3
343	-0.957473	257	0.533333333	-0.533333333
342	-0.96253	258	-0.21	0.21
341	-0.959766	259	-0.98	0.98
340	-1.08701	260	-1.68	1.68
339	-1.1126	261	-2.346666667	2.346666667
338	-1.05426	262	-3.033333333	3.033333333
337	-1.09242	263	-3.643333333	3.643333333
336	-1.03519	264	-4.23	4.23
335	-1.03309	265	-4.78	4.78
334	-0.984389	266	-5.346666667	5.346666667
333	-0.943077	267	-5.836666667	5.836666667
332	-0.912493	268	-6.3	6.3
331	-0.992414	269	-6.77	6.77
330	-1.05035	270	-7.17	7.17
329	-0.983196	271	-7.543333333	7.543333333
328	-0.890541	272	-7.883333333	7.883333333
327	-0.939083	273	-8.22	8.22
326	-0.928324	274	-8.5	8.5
325	-0.858605	275	-8.753333333	8.753333333
324	-0.769391	276	-8.993333333	8.993333333
323	-0.797737	277	-9.19	9.19
322	-0.767545	278	-9.36	9.36
321	-0.836879	279	-9.503333333	9.503333333
320	-0.862586	280	-9.63	9.63
319	-0.927967	281	-9.726666667	9.726666667
318	-0.868056	282	-9.796666667	9.796666667
317	-0.859479	283	-9.846666667	9.846666667
316	-0.813333	284	-9.87	9.87
315	-0.717803	285	-9.88	9.88

314	−0.592677	286	−9.86	9.86
313	−0.499925	287	−9.826666667	9.826666667
312	−0.237821	288	−9.776666667	9.776666667
311	−0.165964	289	−9.713333333	9.713333333
310	−0.127544	290	−9.623333333	9.623333333
309	−0.313647	291	−9.53	9.53
308	−0.393351	292	−9.42	9.42
307	−0.433468	293	−9.3	9.3
306	−0.500866	294	−9.16	9.16
305	−0.446397	295	−9.02	9.02
304	−0.36099	296	−8.87	8.87
303	−0.262501	297	−8.7	8.7
302	−0.0407655	298	−8.53	8.53
301	0.0307064	299	−8.36	8.36
300	0.150267	300	−8.183333333	8.183333333
299	0.0991519	301	−7.99	7.99
298	0.0208866	302	−7.803333333	7.803333333
297	−0.0546081	303	−7.616666667	7.616666667
296	−0.0900994	304	−7.413333333	7.413333333
295	−0.128168	305	−7.22	7.22
294	−0.201321	306	−7.026666667	7.026666667
293	−0.278024	307	−6.833333333	6.833333333
292	−0.289157	308	−6.623333333	6.623333333
291	−0.338937	309	−6.43	6.43
290	−0.236711	310	−6.236666667	6.236666667
289	−0.30884	311	−6.033333333	6.033333333
288	−0.510283	312	−5.843333333	5.843333333
287	−0.544488	313	−5.656666667	5.656666667
286	−0.639191	314	−5.473333333	5.473333333
285	−0.696648	315	−5.276666667	5.276666667
284	−0.655539	316	−5.1	5.1
283	−0.642424	317	−4.92	4.92
282	−0.650911	318	−4.736666667	4.736666667
281	−0.704149	319	−4.57	4.57
280	−0.719907	320	−4.4	4.4
279	−0.862675	321	−4.24	4.24
278	−0.992829	322	−4.07	4.07
277	−1.14735	323	−3.92	3.92
276	−1.37148	324	−3.77	3.77
275	−1.41634	325	−3.61	3.61
274	−1.43546	326	−3.47	3.47
273	−1.46008	327	−3.33	3.33
272	−1.53038	328	−3.2	3.2
271	−1.58481	329	−3.06	3.06

270	-1.62343	330	-2.94	2.94
269	-1.57065	331	-2.816666667	2.816666667
268	-1.53237	332	-2.69	2.69
267	-1.43515	333	-2.58	2.58
266	-1.3783	334	-2.47	2.47
265	-1.24934	335	-2.363333333	2.363333333
264	-1.24315	336	-2.256666667	2.256666667
263	-1.30922	337	-2.16	2.16
262	-1.20513	338	-2.063333333	2.063333333
261	-0.999078	339	-1.966666667	1.966666667
260	-0.611808	340	-1.88	1.88
259	-0.298817	341	-1.796666667	1.796666667
258	0.154228	342	-1.716666667	1.716666667
257	0.579538	343	-1.633333333	1.633333333
256	0.763419	344	-1.563333333	1.563333333
255	1.13581	345	-1.493333333	1.493333333
254	1.48103	346	-1.416666667	1.416666667
253	1.78387	347	-1.353333333	1.353333333
252	2.3312	348	-1.29	1.29
251	3.04769	349	-1.23	1.23
250	3.79416	350	-1.17	1.17
249	4.4207	351	-1.116666667	1.116666667
248	5.17754	352	-1.063333333	1.063333333
247	5.95581	353	-1.01	1.01
246	6.60858	354	-0.963333333	0.963333333
245	7.66842	355	-0.916666667	0.916666667
244	8.71032	356	-0.873333333	0.873333333
243	9.47554	357	-0.83	0.83
242	10.4718	358	-0.79	0.79
241	11.3249	359	-0.75	0.75
240	12.2792	360	-0.71	0.71
239	13.3973	361	-0.68	0.68
238	14.3537	362	-0.646666667	0.646666667
237	15.0414	363	-0.613333333	0.613333333
236	15.7184	364	-0.58	0.58
235	16.5777	365	-0.553333333	0.553333333
234	17.2534	366	-0.53	0.53
233	17.6009	367	-0.5	0.5
232	18.2395	368	-0.476666667	0.476666667
231	18.6083	369	-0.45	0.45
230	19.0984	370	-0.43	0.43
229	19.4679	371	-0.41	0.41
228	19.4173	372	-0.39	0.39
227	18.9082	373	-0.37	0.37

226	18.2517	374	-0.35	0.35
225	17.6971	375	-0.33	0.33
224	16.716	376	-0.313333333	0.313333333
223	15.4039	377	-0.3	0.3
222	13.9433	378	-0.28	0.28
221	12.1329	379	-0.27	0.27
220	10.4968	380	-0.26	0.26
219	8.56139	381	-0.24	0.24
218	6.50576	382	-0.23	0.23
217	4.39633	383	-0.22	0.22
216	2.16134	384	-0.21	0.21
215	-0.15331	385	-0.2	0.2
214	-3.01752	386	-0.19	0.19
213	-6.133	387	-0.18	0.18
212	-8.70015	388	-0.17	0.17
211	-11.2542	389	-0.16	0.16
210	-12.8194	390	-0.15	0.15
209	-14.6357	391	-0.14	0.14
208	-16.1766	392	-0.14	0.14
207	-16.7987	393	-0.13	0.13
206	-17.5055	394	-0.12	0.12
205	-18.0987	395	-0.12	0.12
204	-18.6583	396	-0.11	0.11
203	-18.0979	397	-0.106666667	0.106666667
202	-16.5951	398	-0.1	0.1
201	-14.3952	399	-0.093333333	0.093333333
200	-11.9968			0
